# EGR3 Inhibits Tumor Progression by Inducing Schwann Cell‐Like Differentiation

**DOI:** 10.1002/advs.202400066

**Published:** 2024-07-07

**Authors:** Cai‐hong Chen, Yang Chen, Yi‐nan Li, Heng Zhang, Xiu Huang, Ying‐ying Li, Zhi‐yang Li, Jing‐xia Han, Xin‐ying Wu, Hui‐juan Liu, Tao Sun

**Affiliations:** ^1^ Tianjin Nankai University State Key Laboratory of Medicinal Chemical Biology and College of Pharmacy Tianjin 300350 China; ^2^ Tianjin International Joint Academy of Biomedicine Tianjin 300450 China

**Keywords:** EGR3, melanoma, mRNA vaccine, schwann cell‐like differentiation

## Abstract

The mechanism and function of the expression of Schwann characteristics by nevus cells in the mature zone of the dermis are unknown. Early growth response 3 (EGR3) induces Schwann cell‐like differentiation of melanoma cells by simulating the process of nevus maturation, which leads to a strong phenotypic transformation of the cells, including the formation of long protrusions and a decrease in cell motility, proliferation, and melanin production. Meanwhile, EGR3 regulates the levels of myelin protein zero (MPZ) and collagen type I alpha 1 chain (COL1A1) through SRY‐box transcription factor 10 (SOX10)‐dependent and independent mechanisms, by binding to non‐strictly conserved motifs, respectively. Schwann cell‐like differentiation demonstrates significant benefits in both in vivo and clinical studies. Finally, a CD86‐P2A‐EGR3 recombinant mRNA vaccine is developed which leads to tumor control through forced cell differentiation and enhanced immune infiltration. Together, these data support further development of the recombinant mRNA as a treatment for cancer.

## Graphical abstract

In vitro, EGR3 induces melanoma cells to differentiate into Schwann cell‐like cells, leading to reduced migration, proliferation, and melanin secretion capability. In vivo, the development of a CD86‐P2A‐EGR3 recombinant mRNA vaccine not only triggers melanoma cells to undergo Schwann cell‐like differentiation but also augments immune infiltration via the NK‐cDCs‐CD8^+^ *T*‐cell axis, ultimately resulting in effective tumor control.

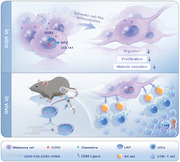

## 1 Introduction

Melanoma is a highly malignant tumor that originates from melanocytes, and melanocytic nevi can exist for decades. Given oncogene‐induced senescence, melanocytic nevi can spontaneously regress, but some may transform into melanoma.^[^ ^1^ ^]^ In ≈25% of melanomas, nevi are observed as precursor lesions.^[^ ^2^, ^3^, ^4^, ^5^ ^]^ Histologically, nevi are classified according to their cytologic maturation into type A, B, and C.^[^ ^6^ ^]^ Type A melanocytes are morphologically similar to normal epidermal melanocytes, are found in the most superficial part of the nevus, and exhibit a nest‐like feature. Type B melanocytes occur in smaller nests in the mid‐dermis and are relatively smaller and round. Type C melanocytes are mainly located in the deep dermis, with more spindle‐shaped cells that produce little or no melanin, accompanied by cell cycle arrest which may represent the last stage of degenerative cell aging. Evidence for the melanocytic origin of dermal nevus cells has been provided by ultrastructural, biochemical, and light microscopic observations.^[^ ^7^, ^8^, ^9^ ^]^ Some type C cells can produce structures that resemble the Wagner–Meissner corpuscles which are known as “nevic corpuscles”. Ultrastructural analysis shows that the type C/ nevic corpuscle region typically contains a complex network of small axons, with collagen deposited in narrow intercellular spaces in the nevus cells.^[^ ^10^, ^11^ ^]^ As early as the 1980s, the type C/ nevic corpuscle region in the deep dermis was observed to possess Schwann cell features and expressed the AHMY‐1 antigen which was identified as myelin protein P0, P1, and myelin basic protein (MBP), indicating that type C nevus cells underwent partial activation of the myelin formation program.^[^ ^12^ ^]^ Although the observation of melanocytes with Schwann cell‐like differentiation in vivo is usually accompanied by the loss of cell migration, proliferation, and melanin production, the driving factors and specific mechanisms of this phenotype transition remain unknown. Interestingly, melanocytes are generally considered to directly originate from neural crest cells, but recent findings suggest that Schwann cell precursors (SCPs) from nerve innervation constitute a cell source for skin melanocytes.^[^ ^13^, ^14^, ^15^, ^16^, ^17^ ^]^ The association between these lineages may imply cross‐gene expression profiles between type C cells and Schwann cells.

Although the characteristics of Schwann cells can be detected in nevi, this phenotype is never adopted in malignant melanoma.^[^ ^18^ ^]^ Even for the neuroendocrine features common in other tumors, their markers CHGA, SYN, and CD56 are considered to have no prognostic significance in melanomas.^[^ ^19^ ^]^ Clinically, rare melanocytic tumors such as melanocytic schwannomas and melanocytic nerve sheath tumors are usually benign,^[^ ^20^, ^21^ ^]^ although their origin remains controversial. Given the lack of reports on the neural lineage differentiation characteristic of melanocyte progenitors — neural crest cells or Schwann precursor cells involved in melanoma initiation and progression — it is tempting to speculate that this phenotype is not conducive to increased melanoma malignancy.

In vitro, B16 cells exposed to IL‐6 and its soluble IL‐6 receptor or IL6RIL6 chimeric protein undergo transdifferentiation from melanoma to a glial cell phenotype.^[^ ^22^, ^23^, ^24^ ^]^ In vivo experiments using B16 cells expressing IL‐6 and its soluble receptor have shown weak subcutaneous tumorigenicity and lung metastasis ability, as well as long survival.^[^ ^25^ ^]^

The above studies suggest a potential negative effect of the glial cell phenotype on melanoma and nevi cells. However, the deeper underlying causes of these phenotypic associations remain unknown. In this study, we used a multi‐round migration screening model and a dropout screening model to identify the transcription factor EGR3, which plays a crucial role in cell migration. EGR3 induces Schwann cell‐like differentiation of melanoma cells by simulating the process of nevus maturation, which leads to a strong phenotypic transformation of the cells, including the formation of long protrusions and a decrease in cell motility, proliferation, and melanin production. We further validated the tumor‐suppressive ability of the EGR3 gene in vivo and through clinical study. Ultimately, the recombinant CD86‐P2A‐EGR3 mRNA vaccine was developed to enhance anti‐tumor immune responses, thereby increasing survival and tumor control.

## 2 Results

### 2.1 Isolation of Low Chemotactic Cells (LCC) Using the Multiple‐Round Migration Screening Model

**Figure 1:**
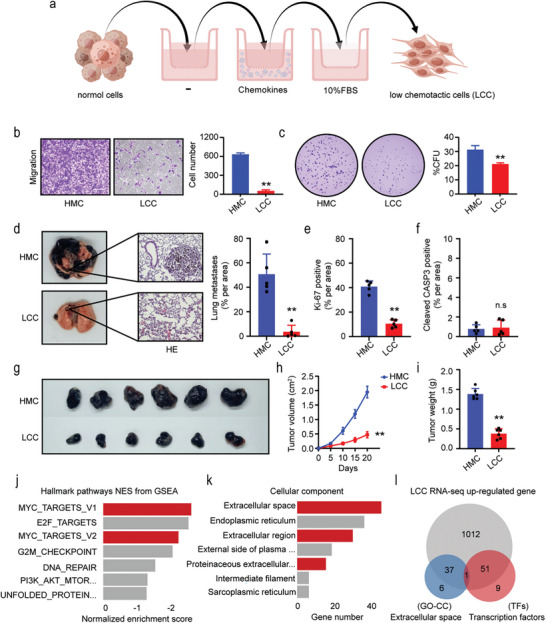
Low chemotactic cells have decreasing malignancy. a) Schematic diagram of the low chemotactic cell sorting model. A375 or B16‐F10 cells were passed through transwell chambers containing a serum‐free concentration gradient medium, a medium for lysophosphatidic acid (LPA) (1 µm) and epidermal growth factor (EGF) (25 ng mL^−1^) chemoattractants, and 10% serum concentration gradient, respectively. b,c) Transwell migration b) and clone formation c) assays were performed in B16‐F10 cells. Scale bars, 100 µm.  d) Representative images of the lungs and of H&E‐stained lung sections from mice that underwent in vivo metastasis assay with B16‐F10 tumor. Scale bars, 200 µm. e,f) Ki‐67 e) and Cleaved CASP3 f) immunostaining and quantitative analysis of positive area in the lung sections with B16‐F10 tumor. g) The tumor formation capabilities were tested by in vivo tumor formation assay with B16‐F10 tumor. h–i) The volume h) and weight i) of B16‐F10 tumor in mice were examined. j,k) Hallmark pathway j) and Gene Ontology‐Cellular Component (GO‐CC) pathway k) enrichment for differential genes from B16‐F10 LCC transcriptome sequencing. l) Venn diagram of up‐regulated genes, extracellular space pathway of differentially expressed genes, and differentially expressed transcription factors from B16‐F10 LCC transcriptome sequencing. Data in (b, h and i) are presented as means ± SEM, *n* = 6. Data in (c) are presented as means ± SEM, *n* = 3. Data in (d,e,f) are presented as means ± SEM, *n* = 5. Statistical significance was determined by Mann–Whitney test b–f,i) and two‐way ANOVA type followed by Tukey's post test h). n.s, not significant; ^**^ *p* < 0.01.

We designed a migration screening model of multi‐round transwell migration using different chemotactic conditions to select cells with low chemotactic (**Figure** **1**a) and highly migratory (Figure S1a, Supporting Information). Through three consecutive rounds of sorting in transwell chambers under varying chemotactic conditions, cells were isolated and categorized as low chemotactic cells (LCC) and highly migratory cells (HMC) based on their migratory behavior. Both A375 and B16‐F10 LCC showed poor migration and weaker proliferation compared to HMC after sorting for low migration ability (Figure 1b,c; Figure S1b,c, Supporting Information). We further examined the lung metastasis and subcutaneous tumorigenicity of B16‐F10 LCC in mice and confirmed a significant reduction in lung metastasis and tumor burden (Figure 1d,g), correlated with decreased proliferation (Ki‐67) rather than increased apoptosis (cleaved caspase‐3) (Figure 1e,f) and decreased tumor volume and weight (Figure 1h,i). We performed transcriptome sequencing of B16‐F10 LCC (Figure S1d, Supporting Information). The reduction of cell proliferation ability was further validated by pathway analysis (reduction of proliferation‐related pathways such as MYC) (Figure 1j). The extracellular space was one of the most significant pathways (Figure 1k; Figure S1e, Supporting Information), suggesting that extracellular components contribute to the cellular features of LCC. In the differentially expressed genes (DEGs) of LCC, both extracellular space‐related genes and transcription factors are predominantly up‐regulated (Figure 1l).

### 2.2 EGR3 drives LCC and Induces Cell Phenotype Transformation

**Figure 2:**
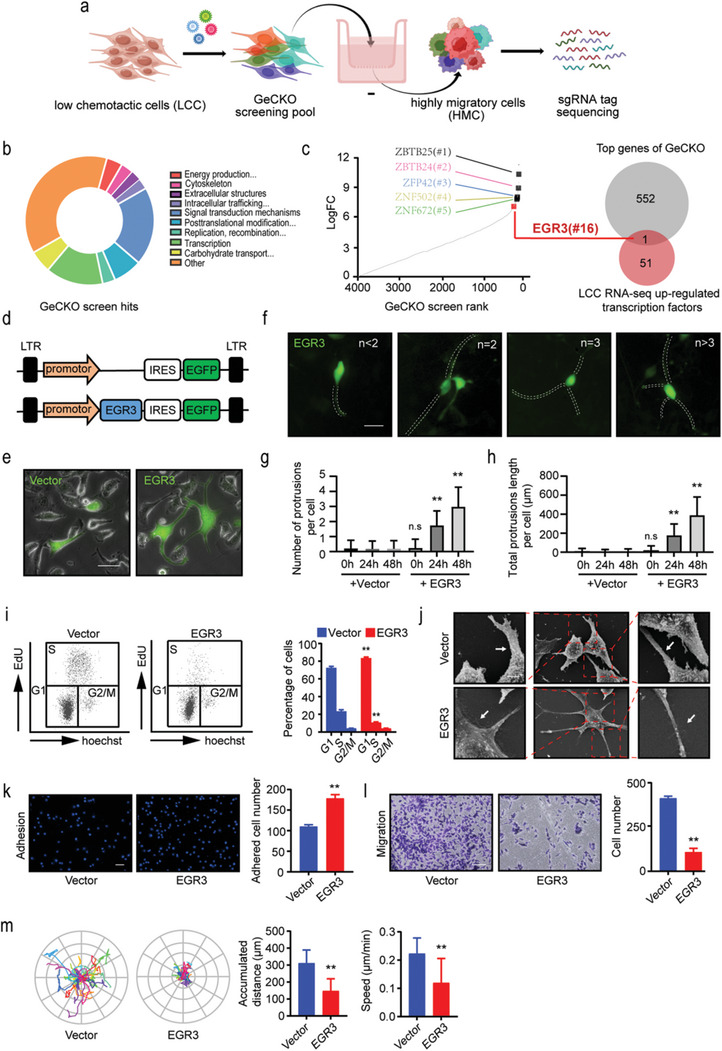
Transcription factor EGR3 drives cells with low chemotaxis. a) Schematic diagram of in vitro dropout screening model in A375 low chemotactic cells. b) Classification of known functions of the differential genes identified in the combined screen ranking from GeCKO‐LCC. c) Distribution of screen scores, demonstrating that only the top genes are enriched. EGR3 was the top gene related to B16‐F10 LCC RNA‐seq up‐regulated transcription factors. d) Schematic diagram of the EGR3 overexpression plasmid construction. e) Overlay images of bright‐field and fluorescence of EGR3‐overexpressing B16‐F10 cells. Scale bars, 20 µm. f–h) Overexpressed EGR3 B16‐F10 cells with different numbers and length of long protrusions. Fluorescence images of B16‐F10 cells overexpressing EGR3 with varying numbers of protrusions f). Quantification of protrusions number g). Quantification of total protrusions length h). The quantification of protrusion number and length commenced 24 h post‐transfection. Scale bars, 20 µm. i) Cell cycle of overexpressed EGR3 B16‐F10 cells detected by flow cytometry analysis. j) Scanning electron microscopy of overexpressed EGR3 B16‐F10 cells. The area within the red dashed box is magnified, with high electron density regions indicated by white arrows. Scale bars, 2 µm. k,l) Representative images of B16‐F10 cells overexpressing EGR3 in the cell adhesion k) and migration experiments l). Scale bars, 100 µm. m) Motion trajectory of overexpressed EGR3 B16‐F10 cells. Data in (g,h) are presented as means ± SEM with *n* = 120. Data in (i) are presented as means ± SEM, *n* = 3. Data in (k and l) are presented as means ± SEM, *n* = 6. Data in (m) are presented as means ± SEM, *n* = 25. Statistical significance was determined by Mann–Whitney test. n.s, not significant; ^**^ *p* < 0.01.

To further explore the driving factors of the LCC, we employed the Genome‐Wide CRISPR Knockout (GeCKO) screening system. This system integrates single‐guide RNAs (sgRNAs) with the endonuclease Cas9. Through the synthesis of specific sgRNAs, Cas9 can be precisely directed to genomic sites, inducing cleavage in nucleotide chains and generating DNA double strand breaks. This process ultimately leads to the destruction and knockout of target genes. Utilizing sgRNA libraries, we conducted CRISPR knockouts on the entire genome, enabling the subsequent screening of target genes.^[^ ^26^, ^27^, ^28^ ^]^ We infected A375 LCC with a GeCKO lentivirus library and screened using a dropout screening model with culture conditions without concentration gradient differences. The GeCKO‐LCC that migrated through the transwell were isolated, amplified, and sequenced to identify enriched sgRNAs (**Figure** **2**a). Genes were scored using an algorithm to calculate the enrichment level of sgRNAs in the separated cells, allowing for the identification of genes that can broadly restrict migration. Many of the top‐scoring genes were involved in cell motion processes, including energy acquisition, cell skeleton, and extracellular structure, a condition that supported the effectiveness of the screening (Figure 2b). Transcription factors have the basic function of controlling gene expression, so we compared the transcription factors enriched through transcriptome sequencing in the LCC transcriptome (Figure 1l) with the 552 genes enriched by GeCKO screening. We found that transcription factor EGR3 was in the intersection of the two types (those enriched by GeCKO screening or by LCC transcriptome sequencing) (Figure 2c). EGR3 belongs to the early growth response (EGR) family of C2H2 zinc finger proteins and plays a role in a variety of processes, including muscle development, lymphocyte development, and neuron development. EGR3 is significantly up‐regulated in both B16‐F10 and A375 LCC (Figure S2a,b, Supporting Information). Rescue of cell migration and proliferation capabilities was achieved by knocking out EGR3 in LCC (Figure S2c–h, Supporting Information). We further constructed an overexpression plasmid with an EGFP label to observe the effects of the EGR3 gene on cell morphology and migration (Figure 2d,e). Interestingly, after EGR3 overexpression, the cells exhibited a morphology with multiple protrusions and an increase in the number and length of cellular protrusions with time, with the average length of protrusions per cell extending by 184.5 µm per 24 h (Figure 2f–h; Figure S2i, Supporting Information). This phenotypic change is accompanied by a decrease in cell proliferation capacity, and cells are arrested in the G1 phase (Figure 2i; Figure S2j, Supporting Information). It is worth mentioning that under electron microscopy observation, the long protrusions exhibit short, filamentous, high electron‐density structures on both sides (indicated by white arrows) (Figure 2j), as well as a regular actin cytoskeleton structure within the protrusions in cell skeleton fluorescent staining (indicated by white arrows) (Figure S2k, Supporting Information). This may contribute to increased cell adhesion ability (Figure 2k), decreased migration (Figure 2l), and reduced motility ability (Figure 2m).

### 2.3 MPZ and COL1A1 are Involved in EGR3‐Induced Schwann Cell‐Like Differentiation of Melanoma Cells

**Figure 3:**
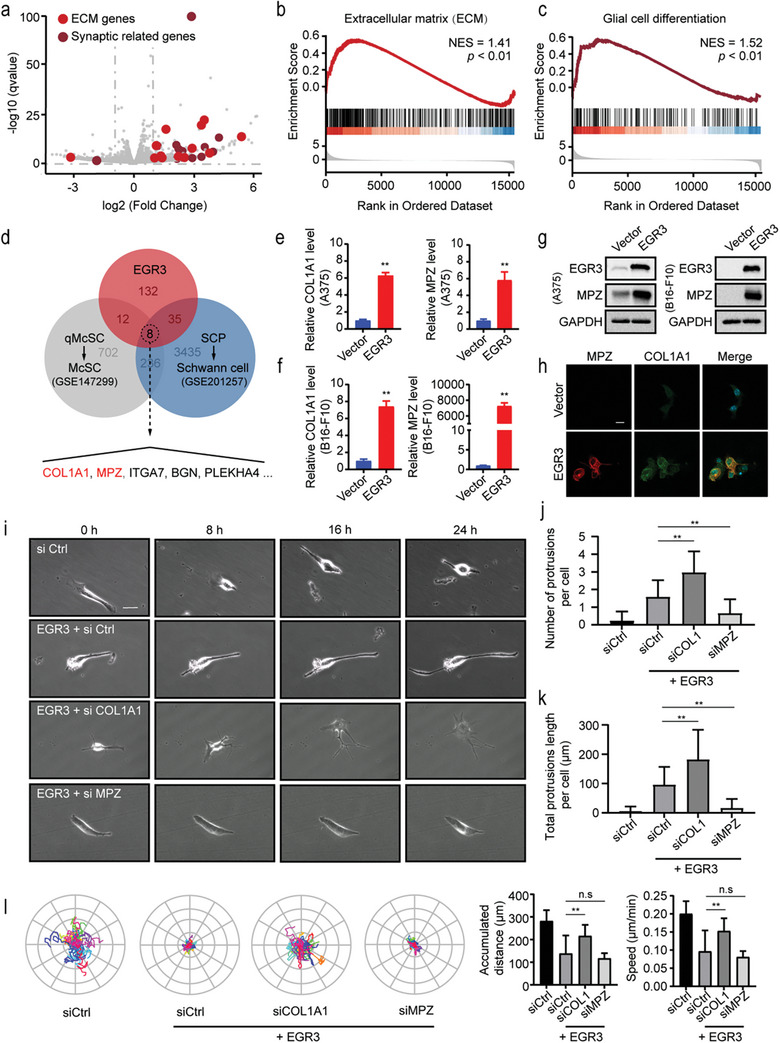
EGR3 induces Schwann cell‐like differentiation of melanoma cells through MPZ and COL1A1. a) Volcano plots presenting the differentially expressed genes between control and overexpressed EGR3 A375 cells. b,c) GSEA from transcriptome sequencing of overexpressed EGR3 A375 cells. d) Venn diagram of differentially expressed genes of EGR3 overexpressed A375 cells, melanocyte differentiation, and Schwann cell differentiation. e–h) The expressions of COL1A1 and MPZ were verified by RT‐qPCR e,f), Western blot g) in EGR3 overexpressed A375 and B16‐F10 cells, and immunofluorescence h) in EGR3 overexpressed B16‐F10 cells. Scale bars, 20 µm. i–k) The effect of knocking down COL1A1 (COL1) and MPZ on long protrusions in overexpressing EGR3 B16‐F10 cells. Representative real‐time imaging of B16‐F10 cells post‐co‐transfection i). Quantification of protrusion numbers in B16‐F10 cells 48 h post‐co‐transfection j). Measurement of total protrusion length in B16‐F10 cells 48 h post‐co‐transfection k). Scale bars, 20 µm. l) Effect of knocking down COL1A1 (COL1) and MPZ on the motion trajectory of overexpressed EGR3 B16‐F10 cells. After 24 h of co‐transfection, the accumulated movement distance and velocity of the cells within 24 h were measured. Data in (e and f) are presented as means ± SEM with *n* = 3. Data in (j and k) are presented as means ± SEM, *n* = 120. Data in (l) are presented as means ± SEM, *n* = 25. Statistical significance was determined by Mann‐Whitney test. n.s, not significant; ^**^ *p* < 0.01.

Transcriptome sequencing of EGR3‐expressing A375 melanoma cells revealed significant up‐regulation of multiple genes associated with the extracellular matrix (ECM), such as COL1A1, COL5A3, and FN1, thereby confirming the regulatory role of EGR3 in the remodeling of the extracellular space. Interestingly, synaptic‐related genes, such as ARC, SYP, and SYT12, were up‐regulated in cells (**Figure** **3**a). Gene Set Enrichment Analysis (GSEA) further enriched pathways related to the extracellular matrix and glial cell differentiation (Figure 3b,c). Note that glial cells, such as astrocytes, oligodendrocytes, and Schwann cells, have protrusion structures. We observed that B16‐F10 and A375 cells overexpressing EGR3 exhibited morphologies similar to those of in vitro cultured Schwann cells. The fact that EGR3 regulates myelin‐related proteins, such as PMP2, PMP22, and MBP, rather than the transcription levels of neuroendocrine markers (Figure S3a, Supporting Information), further supports its association with Schwann cells. The expression of myelin‐related proteins is a primary hallmark of Schwann cell maturation.^[^ ^29^ ^]^ Additionally, we did not observe any regulatory influence on myelin sheath dedifferentiation‐related molecules (Figure S3a, Supporting Information). In recent studies, it has been reported that melanocytes originate from additional Schwann cell precursors.^[^ ^13^, ^14^, ^15^, ^16^, ^17^ ^]^ This discovery led us to speculate whether EGR3 induces melanoma cells to differentiate into Schwann cell‐like cells. As the regulatory mechanism of melanocyte differentiation remains unclear, we cannot exclude the possibility that this phenotype change is involved in normal melanocyte differentiation. However, Schwann cell‐like features have been detected during the maturation of nevi. We compared the overlap of DEGs between EGR3‐overexpressing melanoma cells and the gene expression profiles associated with differentiation in Schwann cells and melanocytes. Eight genes are found within the intersection of the three, these genes may be involved in the development of shared characteristics following the differentiation of SOX10^+^ lineage cells. (Figure 3d).

Due to the positioning of COL1A1 and MPZ genes as the top two in this gene list and their known regulation by early growth response 2 (EGR2),^[^ ^30^, ^31^ ^]^ we proceeded with a more detailed analysis of these two genes. The up‐regulation of COL1A1 and MPZ caused by EGR3 overexpression was validated in both A375 and B16‐F10 melanoma cell lines (Figure 3e–g). Immunofluorescence showed that COL1A1 and MPZ had similar cellular localizations (Figure 3h; Figure S3b, Supporting Information). The expression of extracellular matrix and myelin‐related genes is essential for Schwann cell axon extension, myelination, and stability. Therefore, we further constructed siRNA for these two genes to analyze their effects on cellular protrusion formation, extension, and mobility of cells. After overexpressing EGR3 in B16‐F10 cells, live cell imaging revealed that the position of the cells was relatively fixed, and they extended back and forth in a unipolar manner, resulting in a bipolar shape (Figure 3i). Simultaneously knocking down the COL1A1 expression level while overexpressing EGR3 caused cells to form a multipolar shape with an increase in the number and total length of long protrusions. Correspondingly, knocking down MPZ expression level while overexpressing EGR3 resulted in cells that lack protrusion, indicating the importance of MPZ in protrusion formation (Figure 3j,k; Figure S3c, Supporting Information). Meanwhile, reducing the COL1A1 level but not the MPZ partially restored the cell's mobility, indicating that self‐secreted COL1A1 as the extracellular matrix partially restricts cell movement (Figure 3l). Furthermore, the simultaneous knockdown of MPZ and COL1A1 does not further rescue the enhanced cell migration capability caused by COL1A1 knockdown, indicating that the expression of MPZ is not a limiting factor for cell motility (Figure S3d, Supporting Information). As expected, knockdown of COL1A1 also restored the migration capability of B16‐F10 LCC (Figure S3e, Supporting Information). In fact, pathways, including focal adhesion, regulation of actin cytoskeleton, and PI3K‐AKT signaling pathway, are significantly enriched in EGR3 overexpressing cells (Figure S3f, Supporting Information). The limitation in movement and the elongation of protrusions may be partly attributed to the enhanced pFAK^Tyr397^ and pAKT^Ser473^ caused by COL1A1 (Figure S3g, Supporting Information).

### 2.4 EGR3 Regulates the Levels of MPZ and COL1A1 in a SOX10‐Dependent and SOX10‐Independent Manner, Respectively, Through Non‐Strict Conservative Motifs

**Figure 4:**
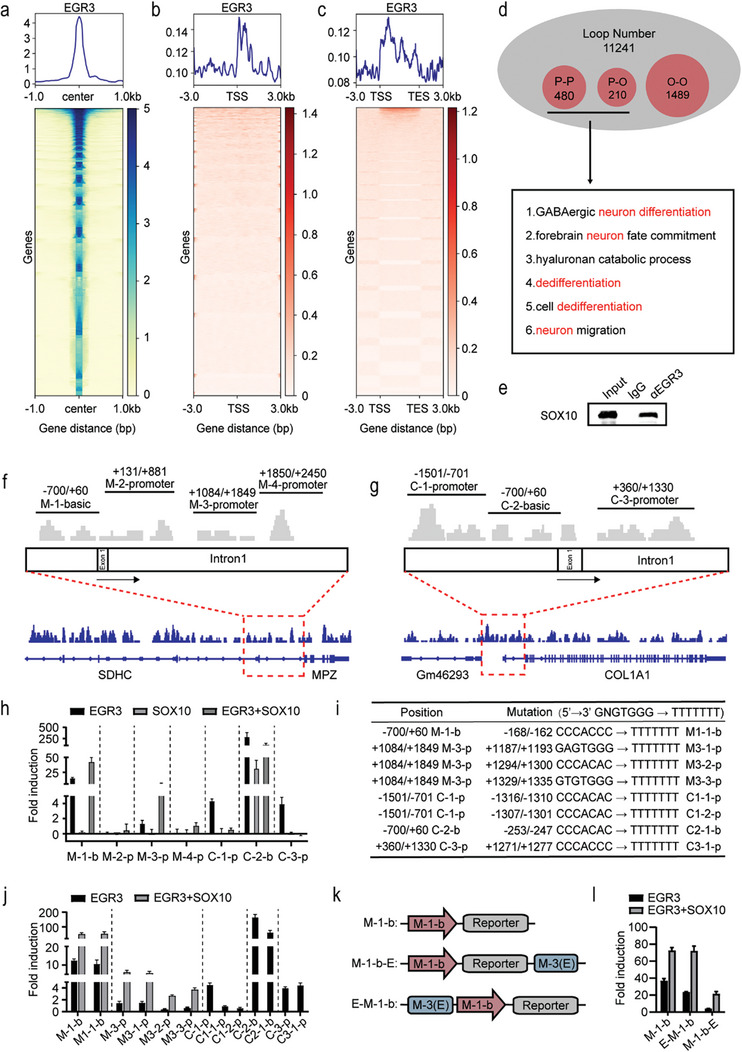
EGR3 regulates MPZ and COL1A1 levels through a SOX10‐dependent and SOX10‐independent manner. a–d) Meta‐peak analysis and heatmaps showing a) the enrichment of EGR3 peaks identified from B16‐F10 LCC, b) the TSS enrichment of EGR3 peaks identified from B16‐F10 LCC, and c) the gene body enrichment of EGR3 peaks identified from B16‐F10 LCC. d) Loop number statistics in the HiCuT assay and pathways significantly enriched in the promoter–promoter loop and promoter–other loop. e) Co‐IP analysis of protein lysates of B16‐F10 LCC by using the EGR3 or the control immunoglobulin G (IgG) antibody and then blotted against SOX10. f,g) Luciferase reporter vectors designed according to the EGR3 binding sites revealed by the CUT&RUN assay. The red dashed box marks the primary binding regions of EGR3 within the MPZ (f) and COL1A1 g) gene loci, and the corresponding truncated segments were cloned into the reporting plasmid. h) 293T cells were co‐transfected with the indicated luciferase reporter vectors with the expression vector for EGR3 and SOX10. Fold induction was calculated relative to the basal level of the empty basic or promoter luciferase reporter. i) Mutant luciferase reporter vectors were further designed according to the previous experimental results. TTTTTTTT was used to replace all 5′→ 3′ GNGTGGG sites in the M‐1‐b, M‐3‐p, C‐1‐p, C‐2‐b and C‐3‐p fragments. j) 293T cells were co‐transfected with the mutant luciferase reporter vectors with expression vector for EGR3 and SOX10. k) The M‐3 (E) fragment was ligated upstream and downstream of the M‐1‐basic reporter vector. l) 293T cells were co‐transfected with the E‐M‐1‐b and M‐1‐b‐E luciferase reporter vectors with expression vector for EGR3 and SOX10. Data in (h, j and l) are presented as means ± SEM with *n* = 3.

The above results suggest that EGR3 may partially drive the differentiation state of melanoma cells. We used HiCuT experiments to detect B16‐F10 LCC, and the analysis of the transcription start site (TSS) and gene body showed that the main peak of EGR3 binding sites is biased toward the downstream of the TSS (**Figure** **4**a–c), especially in the first intron (Figure S4a, Supporting Information). Specific identification of EGR3 established a total of 11241 chromatin loops. Further analysis of the promoter–promoter (P–P) and promoter–other (P–O) chromatin loops showed that EGR3‐mediated loops are mainly involved in neural‐related differentiation and dedifferentiation pathways (Figure 4d). Members of the EGR family are significantly enriched in motif analysis, indicating the conservation of binding sites (Figure S4b, Supporting Information). The motif of SOX10 is also enriched in motif analysis (Figure S4b, Supporting Information). EGR2 collaborates with SOX10 to facilitate the lineage differentiation of Schwann cells and the formation of myelin genes.^[^ ^32^ ^]^ We demonstrate that EGR3 interacts with SOX10 (Figure 4e). Further, reporter gene vectors were designed for the fragment wherein the EGR3 binding peak occurs in the CUT&RUN experiment to verify the main binding sites and activation levels of EGR3 on MPZ and COL1A1 (Figure 4f,g). The 293T cell line was employed for the experiment as it does not express endogenous SOX10, thereby allowing us to observe the synergistic effects between exogenous EGR3 and SOX10. The activating effect of EGR3 was observed in the −700/+60 region and the first intron +1084/+1849 region of the MPZ gene. These two fragments contain multiple conserved elements of SOX10, and the activation is strongly up‐regulated through synergy with SOX10. No individual activating effect of SOX10 was detected in these fragments (Figure 4h). We also observed the activation of EGR3 in the −1501/−701 and −700/+60 region of the upstream of the start of COL1A1 gene and the +360/+1330 region of the first intron, but not in synergy with SOX10 (Figure 4h).

Previous studies have shown that EGR family members bind to the 5′→3′ “GCGTGGG” motif, but further studies on EGR2 in myelin genes confirmed that motifs containing high‐affinity sites for EGR2 are rare, and motifs such as 5′→3′ “GGGTGGG” and “GTGTGGG” are also involved in the EGR2 regulation of myelin.^[^ ^30^ ^]^ Therefore, we further mutated the five fragments with activating effects, and mutated all 5′→3′ “GNGTGGG” motifs in each fragment to “TTTTTTT” motifs to analyze their effects on activation levels (Figure 4i). Mutations at the +1294/+1300 and +1329/+1335 sites in the first intron of MPZ significantly reduced the activation level of EGR3 and its synergistic effect with SOX10 (Figure 4j). Meanwhile, the mutation at the −1316/−1310, −1307/−1301 and −253/−247 site of COL1A1 reduced the activation level of EGR3 (Figure 4j). The +1294/+1300 and +1329/+1335 sites of MPZ, as well as the −1316/−1310 site of COL1A1, have been reported to be regulated by EGR2,^[^ ^31^, ^33^, ^34^ ^]^ in line with the regulatory location of EGR3. This further highlights the similarity of EGR family binding sites.

Interestingly, our investigation into the regions where EGR3 regulates MPZ and COL1A1 unveiled a common motif, “GTGTGGG”. This motif's regulatory effect appears to lack selectivity for genes upstream or downstream. This prompted us to investigate whether EGR3 binding sites could serve as potential enhancer elements. We focused on the “M‐3” fragment within the first intron of MPZ, which harbors EGR3 binding sites that are conserved in humans, mice, and rats.^[^ ^32^ ^]^ To test the enhancer potential of this element, we ligated the “M‐3” sequence upstream and downstream of the M‐1‐b reporter vector, respectively (Figure 4k). Surprisingly, both ligation methods did not enhance the activation of EGR3 for the M‐1‐b reporter vector and instead resulted in a reduction of activity (Figure 4l). Additionally, no further enhancement of synergy with SOX10 was observed (Figure 4l). We hypothesize that EGR3 promotes transcription initiation by altering the extent of duplex opening, either through helicase or topoisomerase activity, and that this enzymatic activity requires stabilization by SOX10. However, further study is needed to confirm this hypothesis.

Notably, it is worth mentioning that this mechanism is not subject to species‐specific influences. HeLa cells, the human cell line, which exhibits characteristic epithelial‐like structural features and does not express SOX10, underwent a morphological transition from regular epithelial‐like to spindle‐shaped cells with protrusion features upon co‐transfection of SOX10 and EGR3 (Figure S4c, Supporting Information). Analysis of the endogenous transcription levels of MPZ and COL1A1 revealed that the sole overexpression of EGR3 upregulated the endogenous COL1A1 gene, but not the MPZ gene. However, upon co‐transfection of EGR3 and SOX10, MPZ exhibited a significant upregulation, providing further evidence for the dependence and independence of SOX10 in regulating MPZ and COL1A1 (Figure S4d, Supporting Information).

### 2.5 EGR3 Exhibits Inhibitory Effects in Clinical and Melanoma Model In Vivo

**Figure 5:**
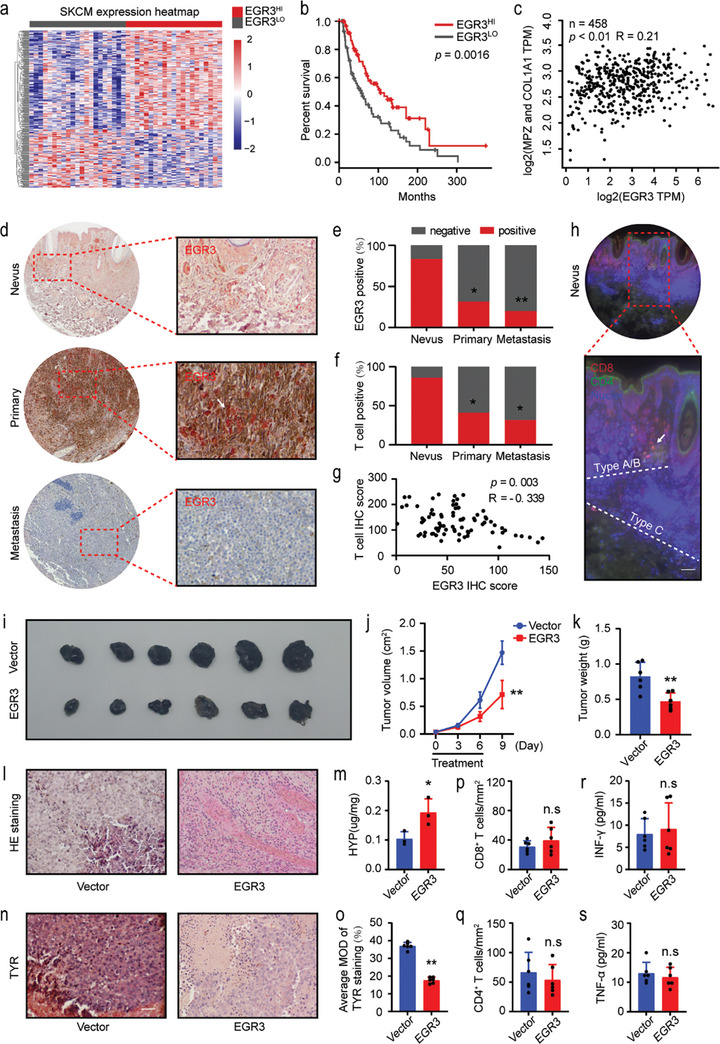
EGR3 slows melanoma progression in vivo. a) Heatmap plot of DEGs in the high EGR3 expression level (EGR3^HI^) patient group versus the low counterpart (EGR3^LO^). b) Kaplan–Meier overall survival curve of TCGA SKCM patients belonging to the EGR3^HI^ or EGR3^LO^ patient groups, *n* = 183. c) Correlation analysis was performed using Spearman correlation analysis, *n* = 458. d) Immunohistochemical staining for EGR3 on nevi, melanoma in situ, and metastatic melanomas from clinical patients. The red dashed box indicates the magnified region. Scale bars, 100 µm. e) Proportion of EGR3 positive sections in immunohistochemical staining from clinical patients. f) Proportion of *T*‐cell positive sections in immunohistofluorescence staining from clinical patients. g) Correlation analysis between EGR3 and *T*‐cells in melanoma in situ from clinical patients. h) CD4^+^ and CD8^+^ *T*‐cells immunohistofluorescence staining in nevi from clinical patients. Two white dashed lines are used to distinguish between Type A/B and Type C nevi regions. The red dashed box indicates the magnified region. Scale bars, 200 µm. i) EGR3 overexpression using adenovirus in subcutaneous B16‐F10 melanoma. j,k) The volume j) and weight k) of the B16‐F10 tumor in mice were examined. l) HE staining of the B16‐F10 tumor tissue. Scale bars, 100 µm. m) The hydroxyproline concentrations were analyzed using a hydroxyproline assay kit. n,o) TYR immunostaining n) and quantitative o) analysis of positive area in the tumor sections. Scale bars, 100 µm. p,q) Quantitative analysis of CD8^+^ p) and CD4^+^ q) *T*‐cell numbers in immunohistofluorescence sections. r,s) Serum IFN‐γ (r) and TNF‐α s) concentrations were measured by ELISA. Data in (j, k, o, p, q, r and s) are presented as means ± SEM with *n* = 6. Data in (m) are presented as means ± SEM, *n* = 3. Statistical significance was determined by chi‐square test e,f), Mann–Whitney test k,m,o,p,q,r,s) and two‐way ANOVA type followed by Tukey's post test j). n.s, not significant; ^*^ *p* < 0.05 and ^**^ *p* < 0.01.

Although preliminary reports indicate a potential association between EGR3 and tumors, the precise biological functions and mechanisms by which EGR3 influences tumor development are still not well understood.^[^ ^35^, ^36^, ^37^, ^38^, ^39^ ^]^ We hypothesize that EGR3, as a tumor suppressor gene, exerts an influence on tumor progression in clinical melanoma patients. Accordingly, we analyzed the DEGs between EGR3^HI^ and EGR3^LO^ patients in the The Cancer Genome Atlas (TCGA) cohort (**Figure** **5**a). Survival analysis showed a positive correlation between EGR3 and survival rates in melanoma cases, and patients with high EGR3 expression had significantly better survival rates than those with low expression (Figure 5b). A positive correlation between the expressions of COL1A1 and MPZ with EGR3 expression in melanoma patients was also observed (Figure 5c). Pathway analysis revealed that EGR3^HI^ patients in melanoma exhibited reduced proliferation signals, such as MYC (Figure S5a, Supporting Information). ECM‐related pathways were significantly enriched in EGR3^HI^ patients, including multiple ECM components such as collagen binding, laminin binding up‐regulated (Figure S5b, Supporting Information). Meanwhile, GSEA, in addition to enriched pathways related to glial cell differentiation, further enriched pathways related to Schwann cell differentiation (Figure S5c,d, Supporting Information).

Meanwhile, we collected and analyzed 87 clinical tissue samples (Table S1, Supporting Information), including 7 nevus and 80 melanomas (39 primary and 41 metastatic lesions). EGR3 expression was observed in dermal nevi (type C region) and some primary melanomas (indicated by white arrows in Figure 5d), with the lowest expression observed in metastatic lesions (Figure 5e). The positivity of *T*‐cells within different regions was further subjected to statistical analysis. The proportion of *T*‐cells gradually decreased in nevi, primary melanomas, and metastatic lesions (Figure 5f). However, further analysis of the 80 melanomas showed a negative correlation between *T*‐cells and EGR3 levels (Figure 5g). In line with this, within nevi, we observed the appearance of *T*‐cells near type B rather than in the type C nevi region (Figure 5h). This suggests that EGR3 may be associated with a reduced immune response. In vitro, EGR3 overexpression in B16‐F10 cells down‐regulated the expression levels of multiple chemokines, including CXCL9, CXCL10, and CXCL11, an outcome which may weaken the chemotactic ability of immune cells (Figure S5e, Supporting Information). As we could not screen for stable overexpression of EGR3 in melanoma cell lines, the adenovirus vector expressing EGR3 was constructed for intratumoral injection of B16‐F10 melanoma in mice (Figure S5f, Supporting Information). Compared to mice injected with the empty vector, those injected with the EGR3 adenovirus exhibited smaller tumor volume and weight (Figure 5i–k) increased collagen content (Figure 5l,m), and decreased expression levels of tyrosinase required for melanin formation (Figure 5n,o). No differences were observed between the two groups in terms of the *T*‐cells in the tumor (Figure 5p,q) or the cytokines IFN‐γ and TNF‐α in their respective sera (Figure 5r,s).

### 2.6 Lipid Nanoparticles (LNPs) Encapsulating Recombinant CD86‐P2A‐EGR3 mRNA Vaccine Mediate Tumor Control of Melanoma

**Figure 6:**
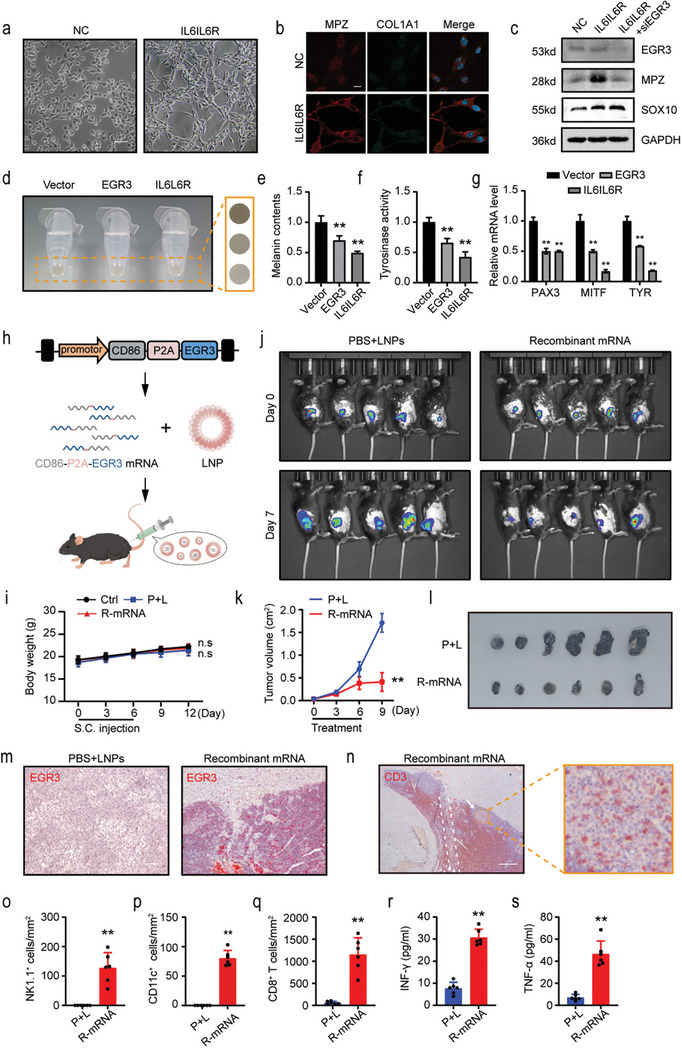
Recombinant mRNA vaccines regress melanoma by enhancing immunity. a) Incubation with IL6IL6R (280 ng mL^−1^) for 48 h induces Schwann cell‐like morphology in B16‐F10 cells. Scale bars, 100 µm. b,c) MPZ expression induced by IL6IL6R depends on EGR3 in B16‐F10 cells. Immunofluorescence images of MPZ and COL1A1 in B16‐F10 cells after induction with IL6IL6R b). Western blot in B16‐F10 cells after induction with IL6IL6R or IL6IL6R plus siEGR3 c). Detection was performed 48 h post‐transfection or addition of IL6IL6R. Scale bars, 20 µm. d–g) EGR3 reduces melanin levels through the PAX3‐MITF‐TYR axis in B16‐F10 cells. Cell pellet image d). Melanin content detection experiment e). Tyrosinase enzyme activity detection experiment f). The mRNA expression levels were assessed using RT‐qPCR experiments g). Detection was performed 48 h post‐transfection or addition of IL6IL6R. The yellow dashed box indicates the magnified region. h) Schematic representation from recombinant CD86‐P2A‐EGR3 plasmid design to recombinant CD86‐P2A‐EGR3 mRNA synthesis and packaging to intratumoral therapy. i) Body weight changes in C57BL/6J mice. Ctrl represents the untreated group, P+L represents the group receiving three subcutaneous injections of PBS plus LNPs, and R‐mRNA represents the group receiving three subcutaneous injections of LNPs encapsulating recombinant mRNA. j) Bioluminescence imaging monitored the B16‐F10 tumor growth between the PBS plus LNPs group and recombinant mRNA group. k) The volume of B16‐F10 tumor in mice between PBS plus LNPs group and recombinant mRNA group was examined. l) Images of tumors in B16‐F10 tumor‐bearing mice after treatment. m) EGR3 immunohistochemical staining after recombinant mRNA vaccine treatment in B16‐F10 tumor. Scale bars, 100 µm. n) CD3 immunohistochemical staining after recombinant mRNA vaccine treatment in B16‐F10 tumor. The white dashed lines indicate the mRNA vaccine injection area, the yellow dashed box indicates the magnified region and the white arrow indicate the *T*‐cells’ attack on the cancer nest. Scale bars, 200 µm. o–q) Quantitative analysis of NK1.1^+^ cell o), CD11c^+^ p) and CD8^+^ *T*‐cell q) numbers in sections of B16‐F10 tumor. r,s) Serum IFN‐γ r) and TNF‐α s) concentrations in B16‐F10 tumor‐bearing mice were measured by ELISA. Data in (e–g) are presented as means ± SEM with *n* = 3. Data in (i) are presented as means ± SEM, *n* = 5. Data in (k, o, p, q, r and s) are presented as means ± SEM, *n* = 6. Statistical significance was determined by Mann–Whitney test e,f,g,o,p,q,r,s) and two‐way ANOVA type followed by Tukey's post test i and k). n.s, not significant; ^**^ *p* < 0.01.

In vivo studies indicate that IL6RIL6 can induce the glial cell transdifferentiation of melanoma cells and inhibit the progression of melanoma.^[^ ^22^, ^23^, ^24^ ^]^ As described earlier, we observed a clear change in the cell phenotype upon addition of IL6RIL6 (**Figure** **6**a). Immunofluorescence showed that IL6RIL6 induced MPZ expression without inducing COL1A1 expression (Figure 6b). Although IL6RIL6 did not increase the expression level of EGR3, MPZ expression was significantly reduced when we knocked down EGR3 (Figure 6c). This outcome may be attributed to the up‐regulation of SOX10 (Figure 6c), which enhances the basal transcriptional activity of EGR3 toward MPZ. Another feature of Schwann cell‐like differentiation in melanoma cells is the degradation of melanin production ability. On the basis of the observed decrease in tyrosinase expression levels in vivo, we further analyzed the effect of EGR3 on melanin production in vitro. Consistent with the IL6RIL6 finding, EGR3 expression resulted in a decrease in tyrosinase activity and cellular melanin levels through down‐regulation of the melanin production pathway of PAX3‐MITF‐TYR (Figure 6d–g).

Recently, mRNA vaccines have generated new directions for tumor treatment. Compared with viral and non‐viral gene therapy vectors, mRNA intratumoral injection vaccines avoid potential risks such as genomic rearrangements, carrier immunity, and backbone effects, which means that these vaccines have great potential in tumor treatment.^[^ ^40^ ^]^ We considered developing an mRNA vaccine that induces Schwann‐cell like differentiation in melanoma cells while simultaneously triggering immune activation. IL6RIL6 has shown favorable clinical efficacy as a whole‐cell melanoma vaccine,^[^ ^41^ ^]^ but it directly activates STAT3 phosphorylation, potentially inhibiting the IFN‐γ‐STAT1 pathway. IFN‐γ‐STAT1 is an important pathway for anti‐tumor immunity after immune infiltration, and the inhibition of its pathway may limit the potential of IL6RIL6 as an mRNA vaccine intratumoral injection for melanoma treatment. By contrast, EGR3 does not directly activate STAT3 phosphorylation (Figure S6a, Supporting Information). To bolster the intratumoral immune response, we introduced the CD86 gene. It has been reported that solitary CD86 exhibits the capability to directly activate NK cells, independent of other co‐stimulatory molecules.^[^ ^42^ ^]^ Concurrently, as a co‐stimulatory signal, CD86 can strengthen the anti‐tumor immune response by enhancing the activity of CD8^+^ *T*‐cells.^[^ ^43^, ^44^ ^]^ Notably, recent findings indicate that NK cells, through the NK‐cDCs‐*T*‐cell axis, play a pivotal role in recruiting *T*‐cells, thereby amplifying the cytotoxic impact on tumors.^[^ ^45^, ^46^ ^]^ In light of these discoveries, we designed a recombinant plasmid, connecting the CD86 and EGR3 genes through the P2A peptide. Utilizing the self‐cleaving property of the P2A peptide within cells, this enabled simultaneous expression of both genes. In vitro, the overexpression of CD86‐P2A‐EGR3 in B16‐F10 cells significantly enhances their interaction with NK cells compared to the control or overexpression of CD86 alone (Figure S6b, Supporting Information). Therefore, we further designed a recombinant CD86‐P2A‐EGR3 mRNA vaccine to induce melanoma cell Schwann cell‐like differentiation and enhance immune response.

We used uridine 5′‐triphosphate with N1‐methylpseudouridine triphosphate (m1ψTP) nucleotides to transcribe the recombinant CD86‐P2A‐EGR3 mRNA. The mRNA is encapsulated with LNPs to enhance the in vivo transfection efficiency (Figure 6h). The standalone subcutaneous injection demonstrates good safety of the vaccine, with no observed abnormal changes in body weight (Figure 6i), biochemical markers (Figure S6c–g, Supporting Information), or organs (Figure S6h, Supporting Information). The recombinant mRNA vaccine effectively controls tumor growth (Figure 6j–l). Further analysis reveals that at the injection site (within the white dashed lines), SOX10‐positive melanoma cells successfully express EGR3 (Figure S7a,b). Treatment with the mRNA vaccine induces extensive immune infiltration (Figure 6m), particularly by *T*‐cells (Figure 6n). Simultaneously, *T*‐cell invasion into the cancer nests can be observed (Figure 6n, white arrow). As anticipated, there is recruitment of NK cells (Figure 6o; Figure S7c, Supporting Information) and DC cells (Figure 6p; Figure S7d, Supporting Information) within the region of recombinant mRNA injection. Outside the injection area, a substantial influx of CD8^+^ *T*‐cells (Figure 6q; Figure S7c, Supporting Information) rather than CD4^+^ *T*‐cells (Figure S7c, Supporting Information) is observed. This is accompanied by elevated levels of IFN‐γ and TNF‐α in the mouse serum (Figure 6r,s). The recombinant mRNA therapy is well‐tolerated in B16‐F10 tumor‐bearing mice, with no observed animal weight loss during the study, resulting in an increased long‐term survival rate (Figure S7e, Supporting Information).

In vivo experiments were further conducted using the highly immunogenic B16‐OVA cell line to investigate whether the efficacy of the vaccine would be impacted by the tumor's intrinsic immune microenvironment. The therapeutic application of recombinant mRNA reduced the number of NK cells in the spleen (Figure S7f,g, Supporting Information) and effectively controlled tumor growth (Figure S7h, Supporting Information). After depleting NK cells using anti‐Asialo‐GM1, there was no difference in tumor volume compared to the group treated with EGR3 mRNA alone (Figure S7h, Supporting Information), indicating the crucial role of NK cells in the initial activation of the immune response to the recombinant mRNA. Simultaneously, there was a significant upregulation of MPZ and COL1A1 levels and a decrease in Ki‐67 levels in the tumor tissue treated with recombinant mRNA, demonstrating the acquisition of Schwann cell‐like characteristics within the tumor (Figure S7i–k). Collectively, these findings illustrate that recombinant mRNA vaccine triggers a Schwann cell‐like differentiation in melanoma cells via the EGR3 gene and bolster immune infiltration through the CD86 gene, ultimately resulting in effective tumor control.

## 3 Discussion

Skin melanocytes have been reported to originate from the SOX10^+^/EGR2^−^ trunk ventral migration pathway of SCPs. Tracking using EGR2^−^Cre/Rosa26^−^YFP confirmed that neurogenic melanocytes do not differentiate from Schwann cells that undergo early myelination and myelin formation in the normal development process,^[^ ^13^ ^]^ indicating relatively independent differentiation pathways of Schwann cells and melanocytes originating from SCPs. BRAF^V600E^ is the most common mutation in nevi and melanoma, and BRAF^V600E^ transgenic mice are commonly used as models for observing nevi. This strain of mice exhibits increased proliferation of skin melanocytes, excessive pigment deposition, and low frequency of melanoma formation. Interestingly, the tumors that form usually resemble neurofibromas, which are benign nerve sheath tumors originating from Schwann cells.^[^ ^18^, ^47^, ^48^ ^]^ Schwann cell‐like tumors in mice rarely develop into malignancy and remain benign throughout the animal's lifespan.^[^ ^18^ ^]^ Multiple studies have also demonstrated a negative correlation between the maturity of nevi and malignant tumors.^[^ ^5^, ^49^ ^]^ The Schwann cell‐like feature may be like a “trap” that nevus cells fall into during the maturation process,^[^ ^18^ ^]^ blocking the migration of melanoma cells with oncogenic mutations deeper into the epidermis, along with cell cycle exit and a decrease in melanin production ability. After EGR3 overexpression in melanoma cells, cell cycle arrest was observed in the G1 phase, accompanied by decreased levels of TYR expression and enzyme activity within the cells, resulting in reduced melanin production.

Senescence may be involved in melanocyte Schwann cell‐like differentiation in vivo. Multiple cell lineages, including melanocytes, have been observed in vitro to undergo RS/OIS (including BRAF^V600E^ and H‐RAS^G12V^) that can lead to degradation of nuclear envelope (NE) proteins.^[^ ^50^, ^51^ ^]^ One effect of NE degradation is the disturbance of the interaction between the genome and the nuclear lamina, resulting in changes in the three‐dimensional chromatin organization in the nucleus.^[^ ^52^ ^]^ In vivo, senescent nevus melanocytes exhibit decreased expression of lamin B1 compared to neighboring keratinocytes and melanocytes.^[^ ^51^ ^]^ Note that according to prior research, during the maturation of neurons, as the expression level of lamin B1 decreases, the genomic locus that contains EGR3 and is located at 14qD2L relocates from the nuclear periphery to the interior, accompanied by the expression of related genes.^[^ ^53^ ^]^ In the skin, lamin B1 expression levels also decrease from the basal layer to the granular layer as keratinocytes differentiate. The EGR3 gene is strongly expressed in skin granular cells, and EGR3 is therefore considered a late epidermal differentiation regulator in the skin.^[^ ^54^ ^]^ In addition, EGR3 is reported to be involved in forced growth arrest and senescence in response to oncogenic stress through the Arf‐Egr‐C/EBPβ pathway.^[^ ^55^ ^]^ All these findings suggest a potential regulatory role of EGR3 in cell maturation/differentiation, which may coordinate with the cell's epigenetic information, ultimately affecting cell function and morphological characteristics. Remarkably, data analysis reveals a significant increase in the expression of the EGR3 gene in melanocytes undergoing differentiation in vivo (GSE147299, data not shown), as well as in melanocytes transfected with BRAF^V600E^ in vitro (GSE46818, data not shown). EGR3 expression in histological sections of clinical patient nevi implies that the regulatory involvement of EGR3 may serve as a universal mechanism for melanocyte maturation and senescence. However, it remains unclear whether in vivo melanocytes undergo NE degradation, which could subsequently impact chromatin spatial remodeling, gene expression changes, and lead to cell differentiation and profound senescence. Further in‐depth studies are needed to elucidate this. Interestingly, another simple and plausible in vivo hypothesis is the BRAF/NRAS mutations, which directly activate the MAPK/ERK pathway and downstream EGR family, leading directly to the occurrence of Schwann cell‐like phenotypes and cell cycle arrest. Therefore, there is reason to suspect that the escape from oncogene‐induced inhibitory differentiation and eventual tumorigenesis inevitably involves the functional transformation of these pathways.

The extracellular matrix is indispensable for Schwann cell myelin sheath formation. The basement membrane provides structural support for anchoring axonal membranes.^[^ ^56^ ^]^ Interaction between Schwann cells and the basement membrane is mediated by layer adhesion protein receptors.^[^ ^57^ ^]^ Additionally, collagen has the capacity to regulate Schwann cell function by modulating intracellular signaling, and it may be crucial for myelin sheath formation.^[^ ^58^ ^]^ EGR family members are important transcription factors involved in extracellular matrix regulation, and EGR1/2 has been reported to regulate the expression of collagen genes such as COL1A1 and COL3A1 through binding to non‐strict conserved regulatory elements in the proximal promoter.^[^ ^31^, ^34^ ^]^ In addition, the EGR family plays an essential role in neurogenesis, with EGR2 involved in regulating the expression of genes related to myelination and maintenance in Schwann cells.^[^ ^59^, ^60^, ^61^ ^]^ In this study, we observed that EGR3 significantly up‐regulates extracellular matrix‐related genes and induces long protrusions on cellular morphology. This outcome is attributable, in part, to the COL1A1 and MPZ genes. Although it remains unclear whether COL1A1 is secreted in the form of a homotrimer or heterotrimer and what arrangement it takes, similar bipolar cellular morphology and restricted cell motility were observed when melanoma cells were cultured on an exogenous type I collagen matrix.^[^ ^62^, ^63^ ^]^ Therefore, we speculate that when trapped by type I collagen, cells extend in a unipolar manner back and forth, producing a cellular morphology similar to that of Schwann cells cultured in vitro.

In this study, we confirmed that EGR3 regulates the expression levels of MPZ by binding non‐strict conserved 5′→3′ “GNGTGGG” motifs, similar to the EGR2. EGR3 regulates the expression of MPZ and COL1A1 in both SOX10‐dependent and ‐independent manners. We observed a closely adjacent pair of non‐strictly conserved motifs with EGR3 regulatory activity in both genetic loci of MPZ and COL1A1. However, it is currently unknown whether this structure can enhance the formation of EGR3 dimers. Further research is needed to examine the regulatory mechanism of this structure. Notably, an inverted orientation and spacing structure similar to the +1084/+1849 intron has been observed in the −5 kb upstream region of MPZ (two reverse EGR sites adjacent to one SOX10 dimer site), which is involved in regulating MPZ transcription levels by EGR2.^[^ ^30^ ^]^ The binding sites of EGR3 appear to only enhance transcriptional activity within specific regions and do not act synergistically as enhancer elements with the transcription start region in vitro. Typically, these active regions are situated upstream of the promoter or within the first intron, and they are frequently spaced apart from one another by a long interval. EGR1 binds to conserved motifs located within the grooves of double‐stranded DNA, using its three zinc finger DNA‐binding domains.^[^ ^64^ ^]^ These domains are conserved among all members of the EGR family, including EGR3.^[^ ^65^ ^]^ Meanwhile, studies on myelin genes have shown that these genes have few high‐affinity EGR2 sites,^[^ ^30^ ^]^ which suggests that the EGR family has evolved a more sophisticated method of regulation. However, the specific mechanism by which the EGR family regulates transcription remains unknown. The similar C2H2‐type member SP1 has been reported to change DNA conformation by DNA unwinding rather than DNA bending.^[^ ^66^ ^]^ Thus, we posit that EGR family members may stabilize the binding of these weaker sites by interacting with the lateral SOX10 dimer, thereby promoting DNA conformational changes and the initiation of transcription. Finally, our study, along with previous research, indicates that as a transcription factor shared by melanocytes and Schwann cells, SOX10 interacts with the EGR family in the form of a complex to participate in gene expression regulation, which is a necessary condition for driving specific lineage differentiation gene transcription.

The development of preventive and therapeutic mRNA strategies has emerged as a novel direction in current cancer management research. Two main approaches, encoding antigens or cytokines, constitute the primary avenues for therapeutic mRNA development in oncology. While mRNA itself has demonstrated sufficient safety, challenges persist in the safety and efficacy of cytokine‐encoding strategies, with concerns about potential systemic immune risks yet to be addressed.^[^ ^67^ ^]^ On the other hand, multiple mRNA cancer vaccines based on tumor‐specific antigens and tumor‐associated antigens have advanced to clinical development stages. However, these two approaches represent distinct active or passive immunotherapy strategies. In contrast, mRNA therapeutic strategies based on tumor cell remodeling hold promise in concurrently addressing both active and passive immunotherapy strategies while reducing the malignant potential of the tumor itself. This may pave the way for new frontiers in mRNA‐based cancer therapy. As a tumor suppressor gene, EGR3 expression in melanoma tissue is associated with longer patient survival. Meanwhile, in vivo experiments demonstrate that EGR3 delays melanoma growth. Due to the potential anti‐proliferative and differentiating capabilities of EGR3, we further developed a recombinant mRNA vaccine for melanoma treatment. CD86 and EGR3 genes were connected using a P2A peptide to prepare a recombinant mRNA vaccine to enhance *T*‐cell activation through NK‐cDCs‐CD8^+^ *T*‐cell axis. Finally, we observed that melanoma tumor‐bearing mice treated with the recombinant mRNA showed an improvement in the survival rate and tumor control. This improvement appears to depend on Schwann cell‐like differentiation and a robust immune response.

LNPs are commonly used carriers for mRNA delivery, but their lack of targeting specificity raises concerns about off‐target toxicity,^[^ ^68^ ^]^ which may limit the routes of administration for recombinant mRNA. Various strategies have emerged to enhance mRNA targeting and reduce side effects, including the modification of LNPs and the use of alternative carriers such as extracellular vesicles, bacterial outer membrane vesicles, and virus‐like particles.^[^ ^69^, ^70^, ^71^, ^72^ ^]^ The development of these strategies promises to improve mRNA targeting while maintaining safety. This provides more options for mRNA‐based cancer therapies via intraperitoneal injection, intravenous administration, and inhalation. For example, in a mouse lung cancer model, inhalation of IL‐12 mRNA encapsulated in extracellular vesicles effectively reduced systemic side effects and enhanced lung targeting.^[^ ^68^ ^]^ The continued development of these strategies holds great potential for expanding the applications of mRNA therapeutics. In conclusion, these data support the further development of recombinant mRNA as a cancer treatment method.

## 4 Experimental Section

### Mice

All animal procedures were approved by the Animal Ethics Committee of Nankai University Laboratory Animal Center (Issue No. 2023‐SYDWLL‐00249). Female C57BL/6J mice were purchased from Charles River. The mice were certified by the Association for Assessment and Accreditation of Laboratory Animal Care. The animals were fed a standard laboratory diet and maintained under a 12 h light/dark cycle. All analyses were double‐blinded and randomized.

### Cell Culture and Treatment

Cells were purchased from KeyGen Biotech (CN) and MeisenCTCC (CN). The murine melanoma cell line (B16‐F10 and B16‐OVA), the human melanoma cell line A375, the human renal epithelial cell line 293T, and the human cervical cancer cell line HeLa were cultured in DMEM (HyClone, USA), supplemented with 10% fetal bovine serum (FBS) (HyClone, USA) and 1% penicillin–streptomycin.

### siRNA and Plasmid Transfection

Cell lines were transfected using Lipo8000 transfection reagent (Beyotime, CN) for the siRNA and plasmid. The concentrations of the siRNA, plasmid, and transfection reagent followed the manufacturer's protocol.

### Quantitative Realtime PCR

RNA was isolated using the Eastep Total RNA Extraction Kit (Promega, CN) and reverse transcribed using 1st Strand cDNA Synthesis SuperMix for qPCR (Yeasen, CN). The accumulation of PCR products was measured by SYBR green fluorescence (Yeasen, CN).

### Western Blot and Co‐Immunoprecipitation

Proteins were extracted using RIPA lysis buffer, and their concentrations were determined via the BCA assay (ThermoScientific, USA). Protein extracts were separated using 10% SDS–polyacrylamide gel and then incubated with primary antibodies (1:1000) at 4 °C overnight. GAPDH (1:5000, Affinity, USA) was used as the loading control, followed by further incubation with horseradish peroxidase‐conjugated secondary antibodies (1:5000, Affinity, USA). Detection was performed using an enhanced chemiluminescence kit (Vazyme, CN). For co‐immunoprecipitation experiments, 50 µL of Protein A/G agarose (Beyotime, CN) was incubated with antibodies against EGR3 overnight at 4 °C with continuous rotation. The lysates were centrifuged at 12 000 rpm for 10 min at 4 °C, and then incubated with the antibody‐conjugated beads overnight at 4 °C. After incubation, the beads were washed three times with a cold lysis buffer. The precipitated proteins were separated from the beads by resuspending the beads in 1×SDS‐PAGE loading buffer and boiling at 99 °C for 10 min. Consequently, the boiled proteins were analyzed by Western blot.

### Multiple‐Round Migration Screening Model and Dropout Screening Model

The melanoma cell lines A375 and B16‐F10 were used in the multiple‐round migration screening model, segregating LCC and HMC in a three‐round migration screening model. Specifically, as illustrated in Figure 1a and Figure S1a (Supporting Information), during the first round, cells were seeded with a cell density of 2 × 10^5^ cells into transwell chambers within six‐well plates. The top and bottom chambers were supplemented with 10% FBS‐DMEM. After 48 h, cells were dissociated using 0.25% trypsin‐EDTA, separating migrated and non‐migrated cells, which were subsequently transferred to 12‐well plates for cultivation and expansion. Upon reaching confluence, the second round of screening was conducted, with cells seeded at a density of 2 × 10^5^ cells into transwell chambers within 6‐well plates. The top and bottom chambers were filled with medium containing 10% FBS‐DMEM, and chemoattractants LPA (1 µm) and EGF (25 ng mL^−1^) were additionally supplied to the bottom chamber's medium.^[^ ^73^ ^]^ After 48 h, cells were dissociated as in the first round, and the screening process was repeated similarly. The third round of screening followed a comparable protocol with cells seeded in transwell chambers. The top chamber was supplied with serum‐free DMEM, while the bottom chamber contained 10% FBS‐DMEM. After 48 h, cells were dissociated using 0.25% trypsin‐EDTA, separating migrated and non‐migrated cells, which were transferred to 12‐well plates for cultivation and expansion, ready for subsequent experiments.

The low chemotactic A375 cells isolated were used in the GeCKO‐mediated dropout screening model. The GeCKO lentivirus library virus was obtained from OBIO Technology (CN). Specifically, as the low chemotactic A375 cells in 6 cm culture dishes reached a 40% confluence, transduction with the GeCKO lentivirus library was carried out at a multiplicity of infection (MOI) of 0.3, and the medium was changed the following day. After 48 h, cells were passaged, and 2 µg mL^−1^ of puromycin was added to the medium. Following a 7‐day puromycin screen, GeCKO library‐transduced LCC cells were used in the dropout screening model. As illustrated in Figure 2a, GeCKO‐LCC cells were seeded with a density of 2 × 10^5^ cells into transwell chambers within six‐well plates. Both the top and bottom chambers were supplemented with 10% FBS‐DMEM. After 48 h, the GeCKO‐HMC cells, which originated from GeCKO‐LCC cells and had migrated to the other side of the transwell membrane, were dissociated using 0.25% trypsin‐EDTA. Subsequently, these cells were transferred to 12‐well plates for cultivation and expansion. GeCKO‐LCC cells that did not undergo dropout screening served as a negative control. Sequencing was performed on DNA extracted from the negative control cells and GeCKO‐HMC cells.

For each sample, guide counts were normalized to the total count of that sample. The counts of guide detected in GeCKO‐HMC cells were compared to the input distribution of guide from negative control to obtain fold‐change values for each guide. The most enriched guide for each gene was identified, and genes were ranked based on their enrichment, with a rank of 1 indicating the highest enrichment. Graphs were generated based on the fold‐change values and ranking of gene.

### Transwell Migration Assay

The experimental methods were as previously described with some modifications.^[^ ^74^ ^]^ Specifically, transwell migration assays were performed by using 8 µm PET transwell (Corning, USA) inserts in 24‐well plates. A total of 1 × 10^4^ cells in serum free‐DMEM were placed in the upper chamber and 10% FBS‐DMEM were added in the lower chamber. After incubation at 37 °C in a humidified 5% CO_2_ atmosphere for 18 h, cells that migrated to another side of the membrane were fixed with methanol, and the non‐migrated cells were mechanically removed with a cotton swab. Cells adherent on the membrane were stained with crystal violet solution. Cell numbers were examined under light microscopy at 200× magnification.

### Single‐Cell Motility Analyses

The experimental methods were as previously described with some modifications.^[^ ^75^ ^]^ For single‐cell motility analyses, the control and treated cells were seeded at 2 × 10^4^ cells in six‐well plates after transfection. Time‐lapse video microscopy images were obtained 24 h after plating, and the cells were manually tracked using the Manual Tracking plugin for the ImageJ software (version 1.49v, Fiji, NIH). At least 20 cells/conditions were tracked in individual experiments.

### Colony Formation and Cell Adhesion Assay

Colony formation assays were used to monitor cellular clonogenic potential. In brief, the control and treated cells were plated in 6‐well plates at 1 × 10^3^ cells/well in triplicate following transfection. After 8 days of incubation, the cells were washed twice with PBS, fixed with methanol for 10 min, stained with 0.1% crystal violet solution for 10 min, and analyzed. For the cell adhesion assay, the cells were plated in six‐well plates at 1 × 10^6^ cells/well. The cells were allowed to adhere for 30 min. Then, the cells were washed twice with PBS and stained with DAPI for 5 min. The number of stained cells per field was counted using a microscope at × 200 magnification.

### Cell Cycle Analysis

For EdU/Hoechst cell cycle analysis, the control and EGR3‐overexpressing B16‐F10 cells were incubated with 10 µm EdU for 2 h at 37 °C. The cells were then fixed with 4% PFA and treated with the EdU Cell Proliferation Kit (Beyotime, CN) according to the manufacturer's instructions. Hoechst 33 342 (Invitrogen, USA) was added at a concentration of 20 µg mL^−1^ for 30 min. The cells were analyzed using BD LSR Fortessa flow cytometer (BD Biosciences, USA), and the data were processed with the FlowJo (version 10) software.

### H&E and Immunohistochemistry

Tumors and multiple organs were fixed in formalin. The tumors and tissues were embedded in paraffin and cut into 5 µm sections. For H&E analysis, the formalin‐fixed paraffin‐embedded tissue slides were stained with H&E for evaluation. For imunohistochemistry analysis, antigen retrieval was performed using citrate‐buffered antigen retrieval solution. The slides were washed and blocked for 30 min with 5% goat serum. The sections were incubated with primary antibodies over night at 4 °C and with secondary antibodies for 1 h at room temperature. Aminoethyl carbazole (AEC) (MXB, CN) was used as the peroxidase substrate. The sections were then washed, counterstained with hematoxylin, mounted, and photomicrographed. Following the previously outlined methodology,^[^ ^76^ ^]^ independent analysis of CD3 and EGR3 expression in sample images was conducted using ImageJ software along with the Colour Deconvolution2 and IHC Profiler plugins.^[^ ^77^, ^78^ ^]^ Scores of high positive (3+), positive (2+), low positive (1+), and negative (0), as well as an overall judgment (positive/negative) were assigned. The H score for each sample, representing the level of EGR3 expression, was calculated using the formula: H score = (High Positive * 3) + (Positive * 2) + (Low Positive * 1).

### Immunofluorescent Staining

Immunofluorescent staining was performed in tissues and cultured cells. Briefly, the cultured cells or tumor sections were fixed in 4% PFA for 15 min and then washed with PBS twice. The samples were permeabilized with blocking buffer (Beyotime, CN) for 30 min at room temperature and then incubated with the indicated primary antibody overnight at 4 °C followed by the fluorescent second antibody (1:200) at room temperature for 2 h. The nuclei were counterstained with DAPI for 30 min, and then sections were mounted on glass and subjected to microscopy. The confocal imaging data were obtained using a Zeiss LSM 800 confocal microscope.

### RNA‐Sequencing

RNA was extracted with the Trizol reagent. Sequencing libraries were generated using the NEBNext Ultra RNA Library Prep Kit for Illumina (NEB, USA) following the manufacturer's instructions. The pooled libraries were sequenced on an Illumina NextSeq sequencer.

### HiCuT and CUT&RUN

HiCuT experiments were completed and analyzed by the Frasergen Bioinformatics Company (CN). CUT&RUN was performed using the CUT&RUN assay kit (CST, USA). Cells were first coated with magnetic beads, followed by permeabilization with digitonin. Antibodies against EGR3 were then incubated with the permeabilized cells. pAG‐MNase enzyme was then added to digest exposed genomic DNA. Finally, the DNA complex was released into the supernatant and was purified for next‐generation sequencing.

### Reporter Assay

Cells were transfected with the indicated reporters and pRL‐TK reporter. At 48 h post transfection, the cells were subjected to the luciferase assay. Luciferase was determined using the Dual‐Lumi Luciferase Assay Kit (Beyotime, CN) by following the manufacturer's instructions.

### Melanin Content and Intracellular Tyrosinase Activity Assay

For the melanin content assay, cells were lysed in 1 n NaOH by heating at 80 °C for 2 h, then absorbance was measured at 405 nm. For the intracellular tyrosinase activity assay, cells were solubilized in a phosphate buffer (0.1 m, pH 6.8) containing Triton X‐100 (0.1%). Cellular lysates were centrifuged at 12 000 rpm at 4 °C for 20 min. The cellular extract was incubated with L‐DOPA (1.25 mm), and the absorbance was identified spectrophotometrically at 475 nm until the reaction was finished.

### NK Cell Selection and Co‐Culture Assay

NK cells were purified using the MagniSort Mouse NK cell Enrichment Kit (Invitrogen, USA) according to the manufacturer's instructions. The NK cells were cultured with the control, EGR3‐overexpressing, CD86‐overexpressing, and EGR3‐P2A‐CD86‐overexpressing B16‐F10 cells at a ratio of 3:1 and were recorded by light and time‐lapse video microscopy.

### Recombinant IL6IL6R Protein, Adenoviruses, and mRNA

Recombinant IL6IL6R protein was purchased from the R&D Systems (USA). Adenoviral reagents Recombinant pcADV‐CMV‐EGR3 and pcADV‐CMV‐MCS adenoviral stocks were obtained from OBIO Technology (CN). Recombinant EGR3‐P2A‐CD86 mRNA was obtained from Azenta Life Sciences (CN). One‐methylpseudouridine (m1Ψ)−5′‐triphosphate instead of UTP was used to generate modified nucleoside‐containing mRNA. The sequence of the CDS region of the recombinant adenovirus and recombinant mRNA is consistent with that of the corresponding plasmid CDS region.

### Encapsulation of mRNA in LNPs

Preparation of aqueous phase: dissolve mRNA powder in deionized water to a concentration of 1 mg mL^−1^. Further, dilute the mRNA solution to 0.17 mg mL^−1^ using a 100 mmol pH 4.0 sodium citrate buffer. The organic phase consists of the FlowOrigin M1 liposomal nanoparticle kit (Mingtai, CN). Load the diluted aqueous phase and organic phase into separate BD syringes, and secure both syringes to the inlets below the FlowTech S microfluidic chip (Mingtai, CN). Synthesis is performed using the MingTai Microflow S microfluidic nanomedicine preparation system (Mingtai, CN).

### Mouse Model Induction and Treatment

Murine melanoma cells were suspended in Dulbecco's phosphate‐buffered saline (DPBS), and 2 × 10^6^ cells in 100 µL were implanted subcutaneously into the right flank of C57BL/6J mice. After 7 days, intratumoral injection was used to administer adenoviruses or mRNA. The mice were anesthetized with 2.5% isoflurane and then intratumorally injected with 5 × 10^8^ pfu adenoviruses or 50 µL mRNA into the right tumor every 3 days for a total of three doses. For the NK cell‐depleted mouse group, on the 6th day after subcutaneous tumor implantation, 100 µL of anti‐Asialo‐GM1 (BioLegend, USA) was intraperitoneally administered. Subsequently, with each recombinant mRNA treatment, 100 µL of anti‐Asialo‐GM1 was intraperitoneally administered.

### Flow Cytometry Analysis

On the second day after the first mRNA treatment, mouse spleens were isolated, homogenized, and passed through a 70‐micron filter to obtain a single‐cell suspension. Cells were then stained with anti‐CD8 Pacific Blue (Invitrogen, USA) and anti‐NK1.1 PE (Proteintech, CN) in the dark at 4 °C for 30 min. The cells were analyzed using BD LSR Fortessa flow cytometer (BD Biosciences, USA), and the data were processed with the FlowJo (version 10) software.

### Cytokine Concentration Analysis

The concentration of IFN‐γ and TNF‐α in mice serum was determined with the mouse IFN‐γ and mouse TNF‐α ELISA Kits (Proteintech, CN).

### Quantifications of Protrusion Number and Length

The number and length of protrusions were manually quantified using ImageJ software. The line tool was employed to measure the length of protrusions of randomly selected B16‐F10 and HeLa cells. Given that B16‐F10 cells exhibited inherent short protrusions, protrusions with a length less than 20 µm were excluded.

### Bioinformatics Analysis

The clinical information and gene expression data of melanoma in TCGA were downloaded using the R package “TCGAbiolinks” and analyzed by the R packages “ggplot2”, “survival” and “survminer”. GSEA was performed using the R package “clusterProfiler”. Patient sample analysis was performed using data from 458 samples in the TCGA melanoma (SKCM) cancer dataset. Groups for EGR3 expression were designated from the top and bottom quintiles of RNA expression.

### Statistical Analysis

Statistical analyses were performed using GraphPad Prism 9 (La Jolla, USA), and the results are expressed as the mean ± SD. Differences between groups were assessed via unpaired two‐tailed *t*‐tests (for simple two‐sample comparison) or one‐way analysis of variance (ANOVA) with Dunnett's test (for multiple comparisons). Kaplan–Meier survival plots and log‐rank tests were used to compare survival between treatment groups. Statistical significance was set at ^*^ *p* < 0.05 or ^**^ *p* < 0.01.

## Conflict of Interest

The authors declare no conflict of interest.

## Supporting information

Supporting Information

## Data Availability

The data that support the findings of this study are available from the corresponding author upon reasonable request.
