# Supplementary material for: EGR3 Inhibits Tumor Progression by Inducing Schwann Cell‐Like Differentiation
Source: Adv Sci (Weinh). 2024 Jul 7;11(34):2400066. doi: 10.1002/advs.202400066 (PMC11425834; doi:10.1002/advs.202400066)
Supplement: Supplementary file 1 — Supporting Information [file ADVS-11-2400066-s001.pdf]

ADVANCED SCIENCE
Open Access

Supporting Information

for *Adv. Sci.*, DOI 10.1002/advs.202400066

EGR3 Inhibits Tumor Progression by Inducing Schwann Cell-Like Differentiation

*Cai-hong Chen, Yang Chen, Yi-nan Li, Heng Zhang, Xiu Huang, Ying-ying Li, Zhi-yang Li, Jing-xia Han, Xin-ying Wu, Hui-juan Liu and Tao Sun**

Supporting Information

EGR3 inhibits tumor progression by inducing Schwann cell-like differentiation

*Cai-hong Chen, Yang Chen, Yi-nan Li, Heng Zhang, Xiu Huang, Ying-ying Li, Zhi-yang Li, Jing-xia Han, Xin-ying Wu, Hui-juan Liu, Tao Sun**

KEY RESOURCES TABLE

REAGENT or RESOURCE	SOURCE	IDENTIFIER
Antibodies		
Mouse monoclonal anti-EGR3	Santa Cruz	Cat# sc-390967;<br>RRID: AB_2894831
Mouse monoclonal anti-EGR3 (Chip grade)	Santa Cruz	Cat# sc-390967 X
Mouse monoclonal anti-COL1A1	Santa Cruz	Cat# sc-59772;<br>RRID: AB_1121787
Rabbit monoclonal anti-MPZ	ZENBIO	Cat# R25059
Rabbit monoclonal anti-SOX10	ZENBIO	Cat# R22801
Rabbit polyclonal anti-STAT3	Affinity	Cat# AF6294;<br>RRID: AB_2835144
Rabbit polyclonal anti-pSTAT3^{Tyr705}	ZENBIO	Cat# 381552
Rabbit monoclonal anti-pSTAT3^{Ser727}	ZENBIO	Cat# R25804
Rabbit polyclonal anti-pan-AKT1/2/3	Affinity	Cat# AF6261;<br>RRID: AB_2835121
Rabbit monoclonal anti-pAKT^{Ser473}	PTM	Cat# PTM-6649
Rabbit monoclonal anti-pFAK^{Tyr397}	ZENBIO	Cat# R22958
Mouse monoclonal anti-FAK	PTM	Cat# PTM-5717
CD8 alpha Monoclonal Antibody (5H10), Pacific Blue™	Invitrogen	Cat# MCD0828;<br>RRID: AB_1488087
anti-Asialo-GM1	BioLegend	Cat# 108907;<br>RRID: AB_2562206
PE anti-mouse NK1.1 (CD161)	Proteintech	Cat# PE-65138;<br>RRID: AB_2883920
Rabbit monoclonal anti-CD11c	Abcam	Cat# ab219799;<br>RRID: AB_2864725

CD3ε (E4T1B) XP® Rabbit mAb	CST	Cat# 78588;<br>RRID: AB_2889902
Alexa Fluor® 647 anti-mouse CD4 Antibody	BioLegend	Cat# 100426;<br>RRID: AB_493519
Alexa Fluor® 488 anti-mouse CD8a Antibody	BioLegend	Cat# 100726;<br>RRID: AB_493423
Donkey Anti-Mouset IgG H&L (Alexa Fluor 568)	Invitrogen	Cat# A10037;<br>RRID:AB_2534013
Donkey Anti-Rabbit IgG H&L (Alexa Fluor 647)	Invitrogen	Cat# A31573;<br>RRID: AB_2536183
Goat Anti-Rabbit IgG (H+L) HRP	Affinity	Cat# S0001;<br>RRID: AB_2839429
Goat Anti-Mouse IgG (H+L) HRP	Affinity	Cat# S0002;<br>RRID: AB_2839430
Biological samples		
Nevus and melanoma samples from patient	Tianjin Medical University general Hospital and<br>Wuhan Bioyeargene	N/A
Chemicals, peptides, and recombinant proteins		
Oleoyl-L-α-lysophosphatidic acid sodium salt	Sigama	L7260
Mouse EGF Recombinant Protein	Gibco	315-09-1MG
hIL-6/IL-6Ra	R&D Systems	8954-SR-025
L-dopa	Aladdin	D111048
Lipo 8000	Beyotime	C0533
Critical commercial assays		
AEC Kit	MXB	AEC-0037
Dual Luciferase Reporter Gene Assay Kit	Beyotime	RG027
EdU Cell Proliferation Kit with Alexa Fluor 555	Beyotime	C0075S
Eastep^{TM} Total RNA Extraction Kit	Promega	LS1030
Hydroxyproline (HYP) Content Assay Kit	Solarbio	BC0250
Mouse IFN-gamma ELISA Kit	Proteintech	KE10001
Mouse TNF-alpha ELISA Kit	Proteintech	KE10002
CUT&RUN assay kit	CST	#86652
Experimental models: Organisms/strains		
C57BL/6J mice	Beijing Vital River Laboratory Animal Technology	

Primers		
Primers for Egr3<br>Forward: CCCATGATTCCCGACTACAA<br>Reverse: TCTTTGAAGGCCTTGATGGT	TsingkeBiotechnology	
Primers for EGR3<br>Forward: GACTCGGTAGTCCATTACAATCAG<br>Reverse: AGTAGGTCACGGTCTTGTTGCC	TsingkeBiotechnology	
Primers for Mpz<br>Forward: CTGCTCCTTCTGGTCCAGTGAA<br>Reverse: AGGTTGTCCCTTGGCATAGTGG	TsingkeBiotechnology	
Primers for MPZ<br>Forward: CTATCCTGGCTGTGCTGCTCTT<br>Reverse: ACTCACTGGACCAGAAGGAGCA	TsingkeBiotechnology	
Primers for Col1a1<br>Forward: CCTCAGGGTATTGCTGGACAAC<br>Reverse: CAGAAGGACCTTGTTTGCCAGG	TsingkeBiotechnology	
Primers for COL1A1<br>Forward: GATTCCCTGGACCTAAAGGTGC<br>Reverse: AGCCTCTCCATCTTTGCCAGCA	TsingkeBiotechnology	
Primers for Mitf<br>Forward: GATCGACCTCTACAGCAACCAG<br>Reverse: GCTCTTGCTTCAGACTCTGTGG	TsingkeBiotechnology	
Primers for Tyr<br>Forward: CAGGCTCCCATCTTCAGCAGAT<br>Reverse: ATCCCTGTGAGTGGACTGGCAA	TsingkeBiotechnology	
Primers for Pax3<br>Forward: GCGTCTCTAAGATCCTGTGCAG<br>Reverse: GATTTCCCAGCTAAACATGCCCG	TsingkeBiotechnology	
Primers for Ki-67<br>Forward: GAGGAGAAACGCCAACCAAGAG<br>Reverse: TTTGTCCTCGGTGGCGTTATCC	TsingkeBiotechnology	
Primers for Gapdh<br>Forward: CATCACTGCCACCCAGAAGACTG<br>Reverse: ATGCCAGTGAGCTTCCCGTTCAG	TsingkeBiotechnology	
Primers for GAPDH	TsingkeBiotechnology	

Forward: GTCTCCTCTGACTTCAACAGCG<br>Reverse: ACCACCCTGTTGCTGTAGCCAA		
siRNA		
siCol1a1-1<br>sense: GAAGAAUACGUAUCACCAA(dT)(dT)<br>antisense: UUGGUGAUACGUAUUCUUC(dT)(dT)	TsingkeBiotechnology	
siCol1a1-2<br>sense: GAGGUAUGCUUGAUCUGUA(dT)(dT)<br>antisense: UACAGAUCAAGCAUACCUC(dT)(dT)	TsingkeBiotechnology	
siCol1a1-3<br>sense: CAAGGUCCUUCUGGAUCAA(dT)(dT)<br>antisense: UUGAUCCAGAAGGACCUUG(dT)(dT)	TsingkeBiotechnology	
siMpz-1<br>sense: GGGAGUCUCGCAAGGAUAA(dT)(dT)<br>antisense: UUAUCCUUGCGAGACUCCC(dT)(dT)	TsingkeBiotechnology	
siMpz-2<br>sense: CCAUUUCGAUCUUCCACUA(dT)(dT)<br>antisense: UAGUGGAAGAUCGAAAUGG(dT)(dT)	TsingkeBiotechnology	
siMpz-3<br>sense: CACGCUCUAUGUCUUUGAA(dT)(dT)<br>antisense: UUCAAAGACAUAGAGCGUG(dT)(dT)	TsingkeBiotechnology	
sgRNA		
KO-EGR3 (A375): CTGCGTACTGAGCGCCCCTG	Genscript Biotech Corporation	
KO-Egr3 (B16-F10): CAGCTTCTCGGCGAGTTTGC	Genscript Biotech Corporation	
Software and algorithms		
IGV	IGV	https://igv.org/
R 4.1.3	R Foundation	https://www.r-project.org/
ImageJ	Open source processing software	https://imagej.nih.gov/ij/
GraphPad Prism 9.0.0	GraphPad	https://www.graphpad.com/scientific-software/prism/
FlowJo 10.6.2 Software	FlowJo LLC	https://www.flowjo.com/
Others		
Zeiss LSM800 with Airyscan	Zeiss	
Zeiss DMi8S	Zeiss	

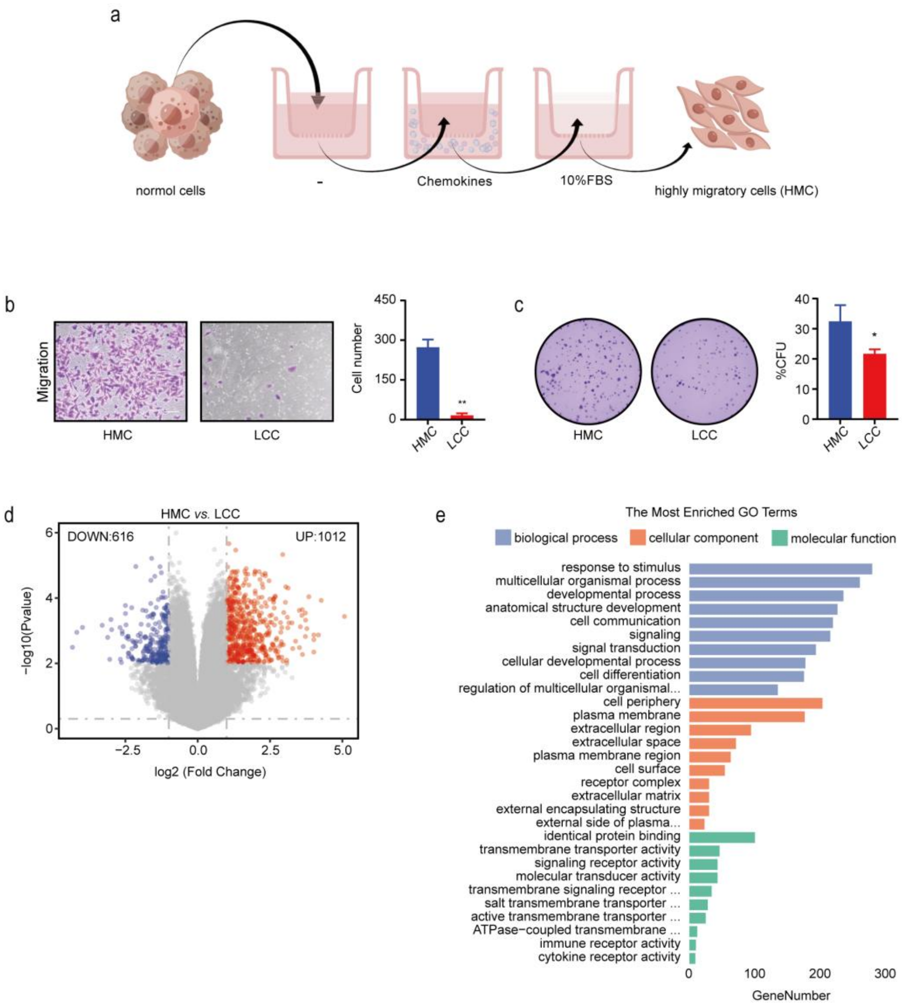
a
normol cells
-
Chemokines
10%FBS
highly migratory cells (HMC)
b
Migration
HMC
LCC
Cell number
450
300
150
0
HMC
LCC
**
c
HMC
LCC
%CFU
40
30
20
10
0
*
d
HMC vs. LCC
DOWN:616
UP:1012
−log10(Pvalue)
log2 (Fold Change)
−2.5
0.0
2.5
5.0
e
The Most Enriched GO Terms
biological process
cellular component
molecular function
response to stimulus
multicellular organismal process
developmental process
anatomical structure development
cell communication
signaling
signal transduction
cellular developmental process
cell differentiation
regulation of multicellular organismal...
cell periphery
plasma membrane
extracellular region
extracellular space
plasma membrane region
cell surface
receptor complex
extracellular matrix
external encapsulating structure
external side of plasma...
identical protein binding
transmembrane transporter activity
signaling receptor activity
molecular transducer activity
transmembrane signaling receptor ...
salt transmembrane transporter ...
active transmembrane transporter ...
ATPase−coupled transmembrane ...
immune receptor activity
cytokine receptor activity
0
100
200
300
GeneNumber

Figure S1 related to Figure 1.

a) Schematic diagram of the highly chemotactic cell sorting model. A375 or B16-F10 cells were passed through transwell chambers containing a serum-free concentration gradient medium, a medium for LPA (1 μM) and EGF (25 ng/ml) chemoattractants, and 10% serum concentration gradient, respectively.

b–c) Transwell migration (b) and clone formation (c) assays were performed in A375 cells. Scale bars, 100 μm.

d) Volcano plots showing Differentially Expressed Genes (DEGs) of HMC vs. LCC in B16-F10 cells.

e) Gene ontology (GO) analyses of up-regulated genes of LCC RNA-Seq data.

Data in (a) are presented as means ± SEM with n = 6. Data in (b) are presented as means ± SEM, n = 3. Statistical significance was determined by Mann-Whitney test. $*p < 0.05$ and $**p < 0.01$.

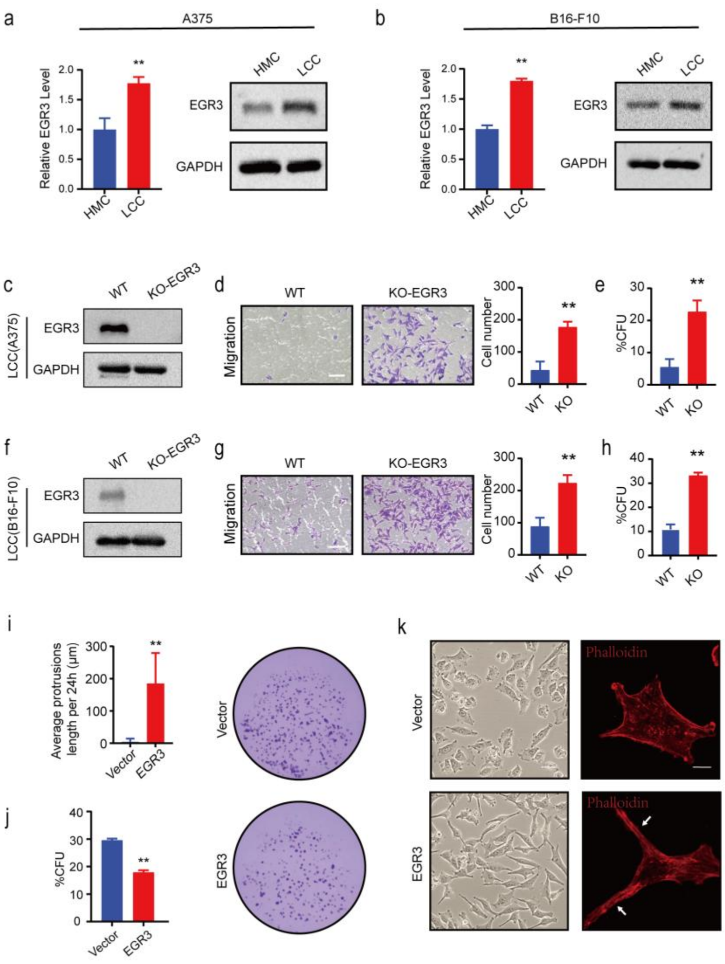
a
A375
Relative EGR3 Level
**
HMC
LCC
EGR3
GAPDH
b
B16-F10
Relative EGR3 Level
**
HMC
LCC
EGR3
GAPDH
c
WT
KO-EGR3
LCC(A375)
EGR3
GAPDH
d
WT
KO-EGR3
Migration
Cell number
**
e
%CFU
**
f
WT
KO-EGR3
LCC(B16-F10)
EGR3
GAPDH
g
WT
KO-EGR3
Migration
Cell number
**
h
%CFU
**
i
Average protrusions length per 24h (μm)
**
Vector
EGR3
j
%CFU
**
Vector
EGR3
k
Vector
EGR3
Phalloidin
Phalloidin

Figure S2 related to Figure 2.

a–b) The expressions of EGR3 in A375 (a) and B16-F10 (b) LCC were verified by RT-qPCR and Western blot.

c–e) The knockout of EGR3 in A375 LCC cells resulted in the restoration of cell migration and proliferation. Western blot reveals EGR3 depletion in A375 LCC (c). Transwell migration (d) and colony formation (e) assays in KO-EGR3 A375 LCC. Scale bars, 100 μm.

f–h) The knockout of EGR3 in B16-F10 LCC cells resulted in the restoration of cell migration and proliferation. Western blot reveals EGR3 depletion in B16-F10 LCC (f). Transwell migration (g) and colony formation (h) assays in KO-EGR3 B16-F10 LCC. Scale bars, 100 μm.

i) The average 24-hour growth length of protrusions in EGR3 overexpressed B16-F10 cells. The average length of protrusions was calculated by quantifying the total length over a 48-hour period, starting 24 hours post-transfection.

j) Clone formation assays were performed in EGR3 overexpressed B16-F10 cells.

k) Representative bright field images and immunofluorescence images after expression of EGR3 in A375 cells. Phalloidin stain is shown in red. Left scale bars, 20 μm. Right scale bars, 10 μm.

Data in (a, b, d, e, g, h and j) are presented as means ± SEM with n = 3. Data in (i) are presented as means ± SEM with n = 120. Statistical significance was determined by Mann-Whitney test. n.s, not significant; *$p < 0.05$; **$p < 0.01$.

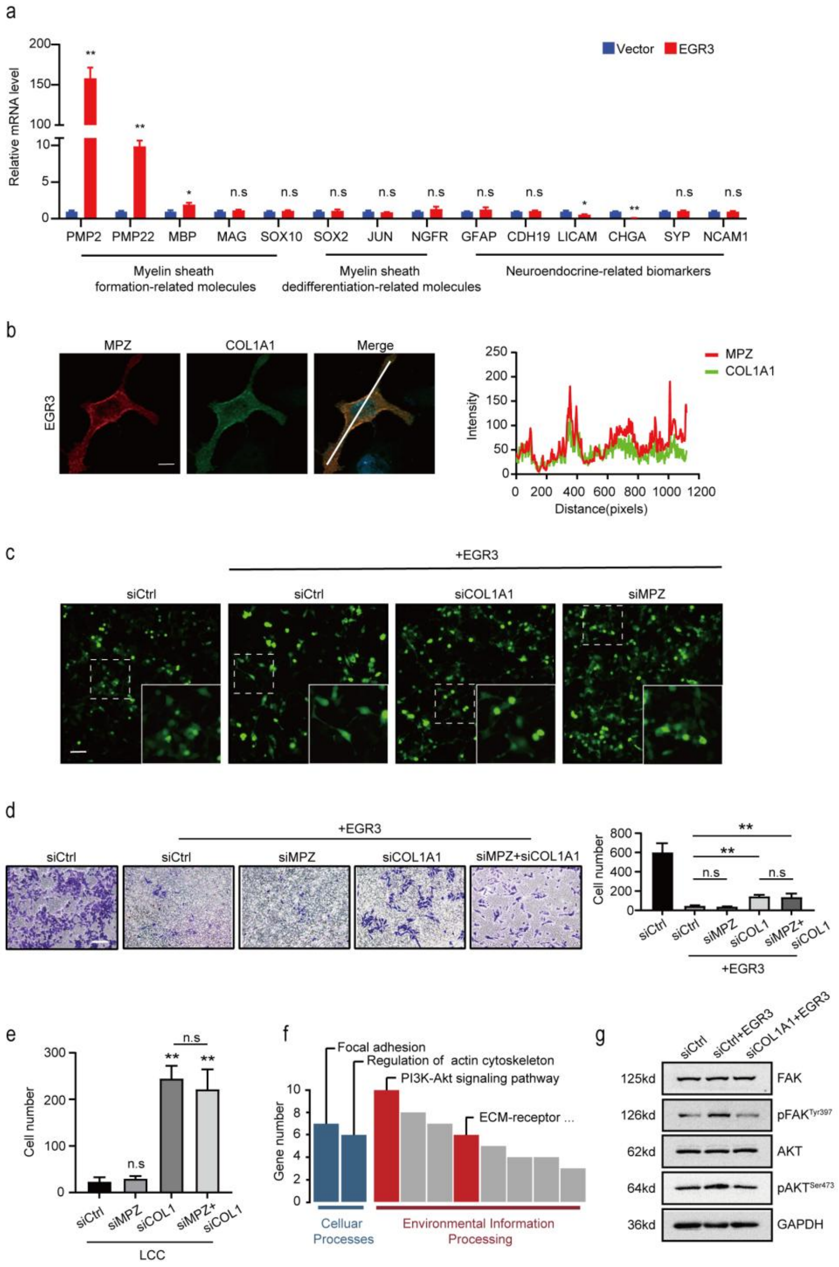
a
Vector
EGR3
Relative mRNA level
PMP2 PMP22 MBP MAG SOX10 SOX2 JUN NGFR GFAP CDH19 LICAM CHGA SYP NCAM1
Myelin sheath formation-related molecules
Myelin sheath dedifferentiation-related molecules
Neuroendocrine-related biomarkers
b
MPZ
COL1A1
Merge
EGR3
Intensity
Distance(pixels)
c
+EGR3
siCtrl
siCtrl
siCOL1A1
siMPZ
d
+EGR3
siCtrl
siCtrl
siMPZ
siCOL1A1
siMPZ+siCOL1A1
Cell number
e
Cell number
siCtrl siMPZ siCOL1 siMPZ+siCOL1
LCC
f
Focal adhesion
Regulation of actin cytoskeleton
PI3K-Akt signaling pathway
ECM-receptor ...
Gene number
Celluar Processes
Environmental Information Processing
g
siCtrl
siCtrl+EGR3
siCOL1A1+EGR3
125kd FAK
126kd pFAKTyr397
62kd AKT
64kd pAKTSer473
36kd GAPDH

Figure S3 related to Figure 3.

a) RT-qPCR analysis of mRNA expression levels in B16-F10 cells overexpressing EGR3.

b) Co-localization analysis of MPZ and COL1A1 in B16-F10 cells overexpressing EGR3. R = 0.9229 (Pearson). Scale bars, 10 μm.

c) Cellular morphology of B16-F10 cells overexpressing EGR3 after transfection with different siRNAs. Scale bars, 100 μm.

d) Transwell migration assay of B16-F10 cells overexpressing EGR3 after transfection with different siRNAs. Scale bars, 100 μm. Scale bars, 100 μm.

e) Transwell migration assay of B16-F10 LCC after transfection with different siRNAs.

f) Annotation results of KEGG for differentially expressed genes were classified by pathway type in KEGG from the transcriptome sequencing of A375 cells overexpressing EGR3.

g) Focal adhesion and PI3K-Akt signaling pathways were validated by Western blot in B16-F10 cells overexpressing EGR3.

Data in (a, d and e) are presented as means ± SEM with n = 3. Statistical significance was determined by Mann-Whitney test. n.s, not significant; $^{**}p < 0.01$.

a

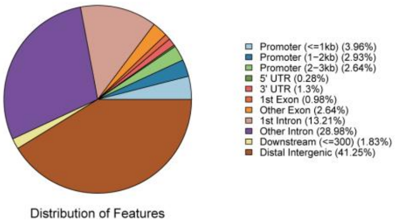

b

Motif	Gene	p-value	E-value
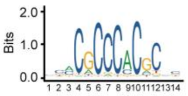	EGR1	7.92e-30	5.91e-27
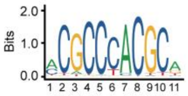	EGR2	2.10e-11	1.57e-8
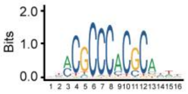	EGR4	9.71e-24	7.24e-21
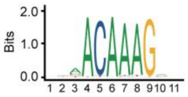	SOX10	1.19e-10	8.84e-8

c

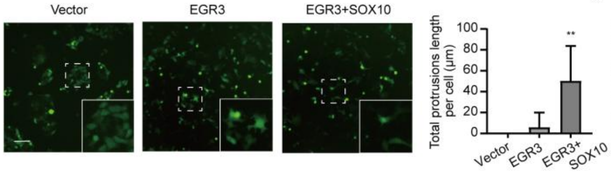

d

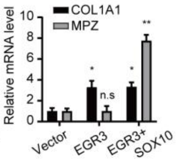

Figure S4 related to Figure 4.

a) Distribution of EGR3 across genomic elements in the HiCuT assay from B16-F10 LCC.

b) Enriched transcription factor binding motifs in the EGR3 binding sites in the HiCuT assay from B16-F10 LCC.

c) Cellular morphology of HeLa cells overexpressing EGR3 or EGR3 plus SOX10. Measurement of total protrusion length in HeLa cells 48 hours post-transfection. Scale bars, 100 μm.

d) The expressions of COL1A1 and MPZ were verified by RT-qPCR in EGR3 or EGR3 plus SOX10 overexpressed HeLa cells.

Data in (c) are presented as means ± SEM with n = 120. Data in (d) are presented as means ± SEM with n = 3. Statistical significance was determined by Mann-Whitney test. n.s, not significant; *$p < 0.05$; **$p < 0.01$.

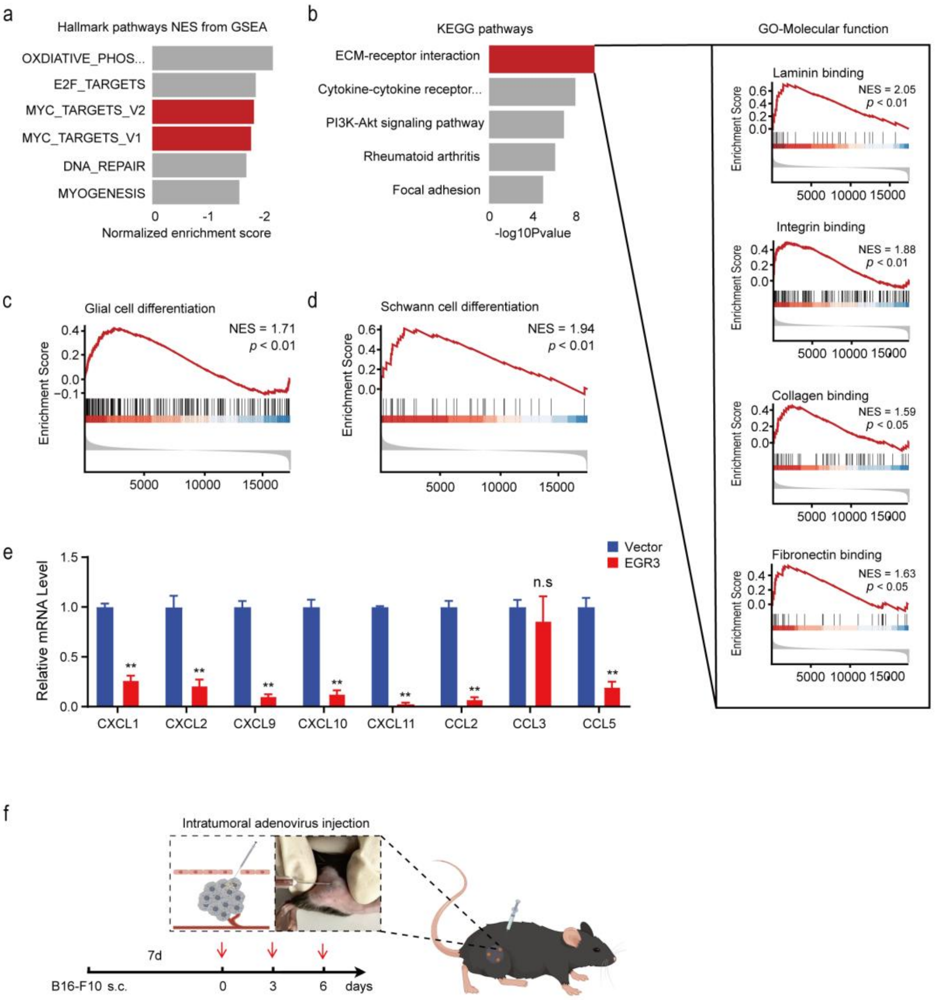
a
Hallmark pathways NES from GSEA
OXDIATIVE_PHOS...
E2F_TARGETS
MYC_TARGETS_V2
MYC_TARGETS_V1
DNA_REPAIR
MYOGENESIS
Normalized enrichment score
b
KEGG pathways
ECM-receptor interaction
Cytokine-cytokine receptor...
PI3K-Akt signaling pathway
Rheumatoid arthritis
Focal adhesion
-log10Pvalue
GO-Molecular function
Laminin binding
NES = 2.05
p < 0.01
Integrin binding
NES = 1.88
p < 0.01
Collagen binding
NES = 1.59
p < 0.05
Fibronectin binding
NES = 1.63
p < 0.05
Enrichment Score
c
Glial cell differentiation
NES = 1.71
p < 0.01
d
Schwann cell differentiation
NES = 1.94
p < 0.01
e
Vector
EGR3
Relative mRNA Level
n.s
CXCL1
CXCL2
CXCL9
CXCL10
CXCL11
CCL2
CCL3
CCL5
f
Intratumoral adenovirus injection
7d
B16-F10 s.c.
0
3
6
days

Figure S5 related to Figure 5.

a–d) GSEA (a, c and d) and KEGG (b) pathway enrichment for differential genes in the high EGR3 expression level ($EGR3^{HI}$) patient group versus the low counterpart ($EGR3^{LO}$) from TCGA SKCM clinical patients.

e) Chemokines expression levels were quantified by RT-qPCR in EGR3 overexpressed B16-F10 cells.

f) The schematic diagram depicts the subcutaneous implantation (s.c.) of B16-F10 cells in C57BL/6J mice for 7 days, followed by intratumoral adenovirus injections administered every 3 days, totaling 3 treatments.

Data in (e) are presented as means ± SEM with n = 3. Statistical significance was determined by Mann-Whitney test. n.s, not significant; $**p < 0.01$.

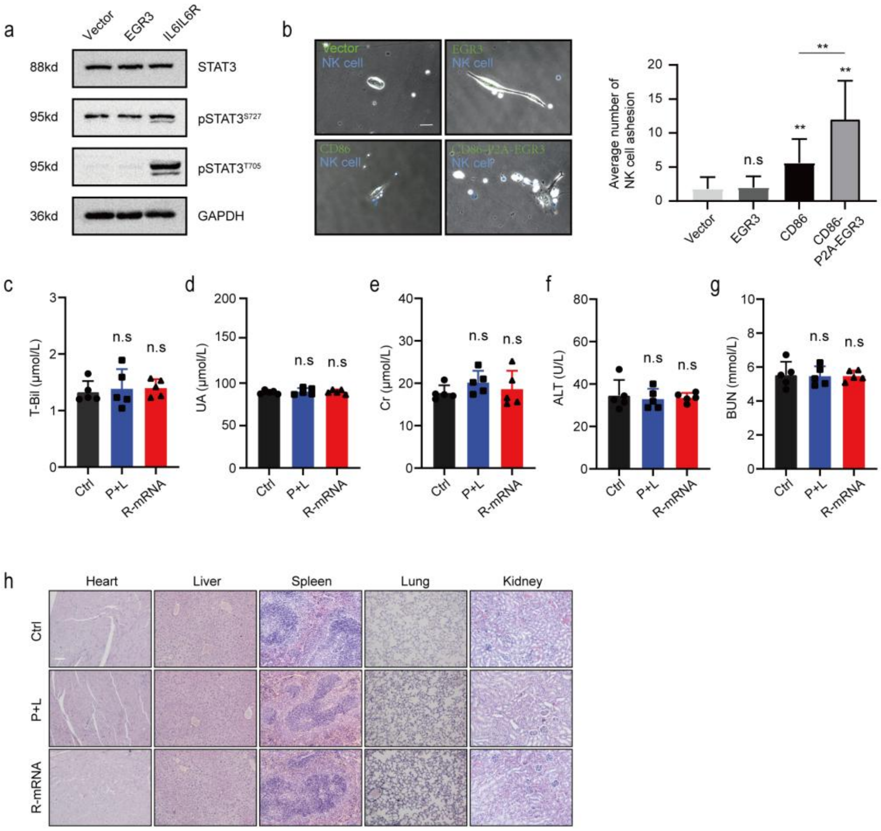
a
Vector
EGR3
IL6IL6R
88kd
STAT3
95kd
pSTAT3S727
95kd
pSTAT3T705
36kd
GAPDH
b
Vector
NK cell
EGR3
NK cell
CD86
NK cell
CD86-P2A-EGR3
NK cell
Average number of NK cell ashesion
20
15
10
5
0
n.s
**
**
**
Vector
EGR3
CD86
CD86-P2A-EGR3
c
T-Bil (μmol/L)
3
2
1
0
n.s
n.s
Ctrl
P+L
R-mRNA
d
UA (μmol/L)
200
150
100
50
0
n.s
n.s
Ctrl
P+L
R-mRNA
e
Cr (μmol/L)
40
30
20
10
0
n.s
n.s
Ctrl
P+L
R-mRNA
f
ALT (U/L)
80
60
40
20
0
n.s
n.s
Ctrl
P+L
R-mRNA
g
BUN (mmol/L)
10
8
6
4
2
0
n.s
n.s
Ctrl
P+L
R-mRNA
h
Heart
Liver
Spleen
Lung
Kidney
Ctrl
P+L
R-mRNA

Figure S6 related to Figure 6.

a) STAT3 and p-STAT3 were validated by Western blot after induction with IL6IL6R or transfection with EGR3 for 48 hours in B16-F10 cells.

b) B16-F10 cells co-cultured with NK cells. Representative images were taken 24 hours after co-culturing. Adhesion quantification was performed 24 hours after co-culturing. Scale bars, 20 μm.

c–g) Total bilirubin (c), uric acid (d), creatinine (e), alanine aminotransferase (f) and blood urea nitrogen (g) levels of C57BL/6J mice on the ninth day.

h) Representative images of various organs in C57BL/6J mice on the ninth day. Scale bars, 500 μm.

Data in (b) are presented as means ± SEM with n = 50. Data in (c–g) are presented as means ± SEM with n = 5. Statistical significance was determined by Mann-Whitney test (b–g). n.s, not significant; ***p* < 0.01.

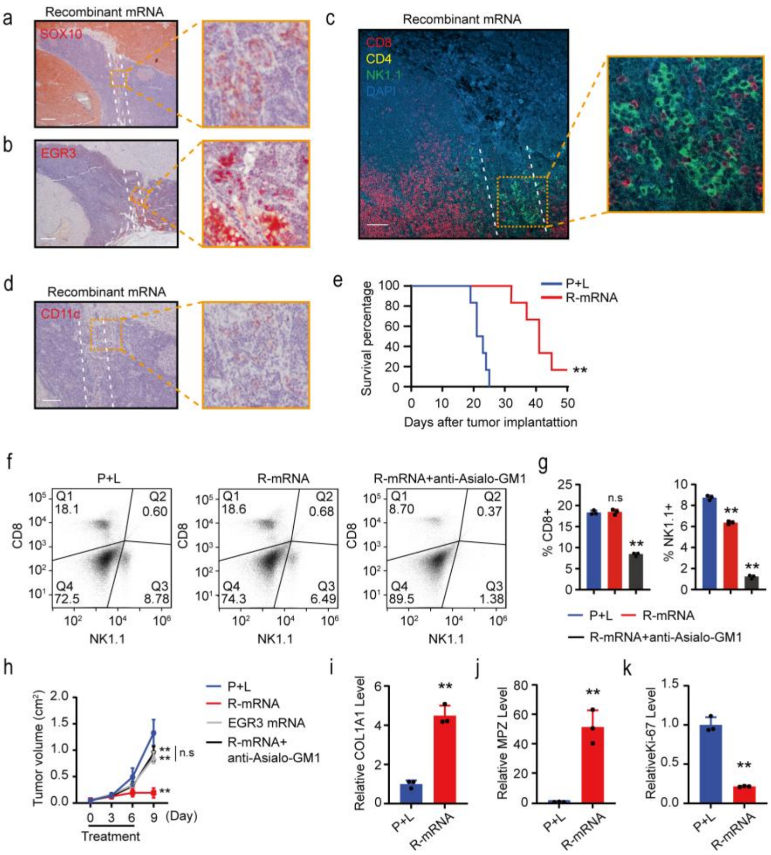

a
Recombinant mRNA
SOX10
b
EGR3
c
Recombinant mRNA
CD8
CD4
NK1.1
DAPI
d
Recombinant mRNA
CD11c
e
P+L
R-mRNA
Survival percentage
Days after tumor implantattion
**
f
P+L
R-mRNA
R-mRNA+anti-Asialo-GM1
CD8
NK1.1
Q1 18.1
Q2 0.60
Q4 72.5
Q3 8.78
Q1 18.6
Q2 0.68
Q4 74.3
Q3 6.49
Q1 8.70
Q2 0.37
Q4 89.5
Q3 1.38
g
% CD8+
% NK1.1+
n.s
**
P+L
R-mRNA
R-mRNA+anti-Asialo-GM1
h
Tumor volume (cm²)
Treatment
(Day)
P+L
R-mRNA
EGR3 mRNA
R-mRNA+
anti-Asialo-GM1
n.s
i
Relative COL1A1 Level
j
Relative MPZ Level
k
RelativeKi-67 Level

Figure S7 related to Figure 6.

a–b) EGR3 (a) and SOX10 (b) immunohistochemical staining of B16-F10 tumor after recombinant mRNA vaccine treatment. The white dashed lines indicate the mRNA vaccine injection area and the yellow dashed box indicates the magnified region. Scale bars, 500 μm.

c) CD8, CD4 and NK1.1 immunohistofluorescence staining of B16-F10 tumor after recombinant mRNA vaccine treatment. The white dashed lines indicate the mRNA vaccine injection area and the yellow dashed box indicates the magnified region. Scale bars, 200 μm.

d) Representative images of $CD11c^{+}$ cells in the immunohistochemical staining of B16-F10 tumor. The white dashed lines indicate the mRNA vaccine injection area and the yellow dashed box indicates the magnified region. Scale bars, 200 μm.

e) Overall survival of mice implanted with B16-F10 tumors is shown. Starting at day 7, mice were treated with three doses of 50 μg of recombinant mRNA, administered every 3 days.

f) B16-OVA melanoma-bearing mice spleen flow cytometry analysis after recombinant mRNA treatment. Anti-Asialo-GM1 was used to deplete NK cells.

g) Analysis of the distribution of $CD8^{+}$ and $NK1.1^{+}$ cell populations in the spleen by flow cytometry in B16-OVA melanoma-bearing mice.

h) The volume of B16-OVA tumor in mice was examined.

i–k) RT-qPCR analysis of COL1A1 (i), MPZ (j) and Ki-67 (k) mRNA expression levels in B16-OVA tumor.

Data in (e and h) are presented as means ± SEM with n = 5. Data in (g, i, j and k) are presented as means ± SEM, n = 3. Statistical significance was determined by Mann-Whitney test (g, i, j and k), two-way ANOVA type followed by log-rank test (e) and Tukey's post test (h). n.s, not significant; $^{**}p < 0.01$.

Table S1. Clinical information of patients. The information about the patient sex, age, and tumour characteristics were listed.

Sex	Age	Diagnosis	TNM
F	15	nevus	-
M	6	nevus	-
M	14	nevus	-
F	8	nevus	-
M	16	nevus	-
M	18	nevus	-
F	66	nevus	-
F	23	melanoma	T3N0M0
M	84	melanoma	T3N2M0
M	25	melanoma	T3N1M0
F	53	melanoma	T1N3M0
M	53	melanoma	T1N0M0
M	29	melanoma	T3N0M0
F	53	melanoma	T2N0M0
F	80	melanoma	T3N2M0
M	64	melanoma	T3N2M0
M	64	melanoma	T3N0M0
F	54	melanoma	T3N0M0
M	59	melanoma	T3N0M0
F	48	melanoma	T2N0M0
M	25	melanoma	T2N3M0
M	11	melanoma	T1N0M0
F	60	melanoma	T2N0M0
M	24	melanoma	T2N0M0
F	82	melanoma	T3N1M0
M	18	melanoma	T2N3M0
F	77	melanoma	T2N0M0
M	79	melanoma	T3N0M0
M	52	melanoma	T3N0M0
M	55	melanoma	T1N0M0
M	48	melanoma	T3N0M0
F	47	melanoma	T3N1M0
F	54	melanoma	T3N0M0
F	75	melanoma	T3N0M0
F	41	melanoma	T3N0M0
M	49	melanoma	T1N0M0
M	65	melanoma	T4N0M0

F	84	melanoma	T4N0M0
M	72	melanoma	T2N0M0
M	46	melanoma	T3N1M0
F	66	melanoma	T3N0M0
F	42	melanoma	T4N0M0
F	72	melanoma	T2N0M0
M	43	melanoma	T3N0M0
F	69	melanoma	T3N1M0
F	69	melanoma	T2N1M0
M	50	Metastatic melanoma	-
F	53	Metastatic melanoma	-
F	48	Metastatic melanoma	-
F	76	Metastatic melanoma	-
M	44	Metastatic melanoma	-
M	45	Metastatic melanoma	-
F	71	Metastatic melanoma	-
M	69	Metastatic melanoma	-
F	51	Metastatic melanoma	-
M	48	Metastatic melanoma	-
M	73	Metastatic melanoma	-
M	57	Metastatic melanoma	-
F	56	Metastatic melanoma	-
M	77	Metastatic melanoma	-
M	62	Metastatic melanoma	-
F	22	Metastatic melanoma	-
F	63	Metastatic melanoma	-
F	48	Metastatic melanoma	-
F	61	Metastatic melanoma	-
F	56	Metastatic melanoma	-
M	47	Metastatic melanoma	-
F	74	Metastatic melanoma	-
F	28	Metastatic melanoma	-
M	74	Metastatic melanoma	-
M	68	Metastatic melanoma	-
M	76	Metastatic melanoma	-
F	60	Metastatic melanoma	-
M	76	Metastatic melanoma	-
M	62	Metastatic melanoma	-
F	48	Metastatic melanoma	-
M	63	Metastatic melanoma	-
M	45	Metastatic melanoma	-
F	64	Metastatic melanoma	-
M	37	Metastatic melanoma	-

M	91	Metastatic melanoma	-
F	68	Metastatic melanoma	-
M	44	Metastatic melanoma	-
F	48	Metastatic melanoma	-
F	61	Metastatic melanoma	-
F	71	Metastatic melanoma	-
F	59	Metastatic melanoma	-

F: female; TNM: Tumor, Node, Metastasis
